# Frauen in der Gastroenterologie – Status quo einer deutschen Umfrage: Warum Abwarten keine Lösung ist

**DOI:** 10.1055/a-2797-1759

**Published:** 2026-03-24

**Authors:** Sandra Nagl, Ana Dugic, Yarema Okhrin, René Barnickel, Ulrike Denzer

**Affiliations:** 1 III. Medizinische Klinik 39694 Universitätsklinikum AugsburgAugsburgBYGermany; 2 Innere Medizin IV 152528 Universität Heidelberg Medizinische Fakultät HeidelbergHeidelbergBWGermany; 3 Lehrstuhl für Statistik und Data Science 26522 Universität AugsburgAugsburgBYGermany; 4 Deutsche Gesellschaft für GastroenterologieDeutsche Gesellschaft für GastroenterologieMarburgHEGermany; 5 Klinik für Gastroenterologie, Endokrinologie und Stoffwechsel 61061 Universitätsklinikum Gießen und Marburg - Standort MarburgMarburgHEGermany

**Keywords:** Geschlechterunterschiede, Gastroenterologie, (interventionelle) Endoskopie, Führungspositionen, Karriereentwicklung, Teilzeitarbeit, Weiterbildung, Gender differences, Gastroenterology, (Interventional) endoscopy, Leadership positions, Career development, Part-time work, Professional development

## Abstract

**Einleitung:**

Trotz eines steigenden Anteils weiblicher Medizinstudierender und Ärztinnen bestehen in der Gastroenterologie und Endoskopie weiterhin geschlechtsspezifische Ungleichheiten, insbesondere in Führungspositionen. Ziel dieser Studie war die Analyse geschlechtsspezifischer Unterschiede sowie wahrgenommener struktureller Barrieren in Deutschland.

**Material und Methoden:**

Im Februar 2025 wurden alle Mitglieder der Deutschen Gesellschaft für Gastroenterologie (DGVS) zu einer anonymen Online-Umfrage mit 16 standardisierten Fragen eingeladen. Erfasst wurden berufliches Tätigkeitsfeld, Ausbildungsstand, endoskopische Tätigkeit und Weiterbildung mit Fokus auf geschlechtsspezifische Unterschiede.

**Ergebnisse:**

685 DGVS Mitglieder nahmen teil. Männer waren signifikant häufiger in leitenden Positionen tätig (15,33% vs. 5,58%, p=0,0002). Frauen arbeiteten häufiger in Teilzeit (43,12% vs. 15,33%, p<0,0001) und führten seltener komplexe interventionelle Endoskopien (p<0,0001) durch. Zudem berichteten sie häufiger über Unzufriedenheit mit ihrer endoskopischen Ausbildung (42,38% versus 20,92%, p<0,0001), insbesondere aufgrund familiärer Verpflichtungen und eingeschränkter Vereinbarkeit von Beruf und Familie.

**Zusammenfassung:**

Die Studie zeigt persistierende geschlechtsspezifische Disparitäten in der Gastroenterologie. Strukturelle Fördermaßnahmen und verbesserte Vereinbarkeitsmodelle sind notwendig, um Chancengleichheit und Karriereentwicklung von Frauen zu stärken.

## Abstract (en)

**Introduction:**

Despite the increasing proportion of female medical students and physicians, gender-specific disparities persist in gastroenterology and endoscopy, particularly with regard to leadership positions. The aim of this study was to analyze gender-related differences and perceived structural barriers in Germany.

**Materials and Methods:**

In February 2025, all members of the German Society for Gastroenterology (DGVS) were invited to participate in an anonymous online survey comprising 16 standardized questions. Data were collected on professional practice acitivity, training status, endoscopic practice, and continuing medical education, with a focus on gender-specific differences.

**Results:**

A total of 685 DGVS members participated. Men were significantly more likely to hold leadership positions (15.33% vs. 5.58%, p=0.0002). Women more frequently worked part-time (43.12% vs. 15.33%, p<0.0001) and performed complex interventional endoscopic procedures less often (p<0.0001). In addition, women reported significantly higher dissatisfaction with their endoscopic training (42.38% vs. 20.92%, p<0.0001), primarily due to family responsibilities and challenges balancing work and family life.

**Conclusion:**

This study demonstrates persistent gender disparities in gastroenterology. Targeted structural interventions and improved models for reconciling professional and family responsibilities are needed to ensure gender equity and support women’s career progression.

## Abkürzungsverzeichnis

x ^2^: Chi-Quadrat
DGVS: Deutsche Gesellschaft für Gastroenterologie, Verdauungs- und Stoffwechselkrankheiten
EMR: endoskopische Mukosaresektion
ERCP: endoskopische retrograde Cholangiopankreatikografie
ESD: endoskopische Submukosadissektion
ESGE: European Society of Gastrointestinal Endoscopy
EUS: endoskopische Ultraschalluntersuchung
GEH: Gastroenterologie und Hepatologie
KI: Konfidenzintervall
ÖGD: Ösophagogastroduodenoskopie

## 1. Einleitung

In den vergangenen Jahrzehnten hat sich der Anteil weiblicher Medizinstudierender in Deutschland kontinuierlich erhöht. Während im Jahr 1975 Männer noch deutlich überwogen, lag der Anteil weiblicher Studierender laut einer Umfrage des *Deutschen Ärzteblatts* im Jahr 2021 bereits bei 73,20%. Im ärztlichen Beruf nähert sich das Geschlechterverhältnis inzwischen einer Parität an – im Jahr 2023 waren nahezu ebenso viele Ärztinnen wie Ärzte tätig 1 . Dieses Bild einer scheinbar erfolgreichen Gleichstellung ernüchtert sich jedoch bei detaillierter Betrachtung der Karrierestufen und Führungspositionen im medizinischen System.

Das sogenannte *Leaky-Pipeline* -Phänomen, das den überproportionalen Verlust von Frauen auf dem Karriereweg beschreibt, ist in der Medizin weiterhin präsent. Trotz hoher Absolventinnenzahlen reduziert sich der Frauenanteil im Verlauf der wissenschaftlichen Laufbahn signifikant – insbesondere zwischen Promotion und Habilitation ist ein Rückgang von etwa 30% zu verzeichnen 2 . Auch wenn Ärztinnen in einigen Fachrichtungen, etwa der Allgemeinmedizin, Pädiatrie und Gynäkologie, dominieren, bleibt ihre Repräsentation in leitenden Funktionen weiterhin deutlich unterproportional. So sind lediglich rund 13% der Spitzenpositionen an deutschen Universitätsklinika mit Frauen besetzt 1 . Der Anteil habilitierter Ärztinnen liegt bei nur 15,5% 1 , und auf C4-Professuren entfällt ein Männer-Frauen-Verhältnis von 4:1 3 . Eine Erhebung aus dem Jahr 2021 ergab, dass an 33 von 34 teilnehmenden Universitätsklinika keine einzige Frau als Klinikdirektorin tätig war 4 . Eine aktuelle Auswertung von 36 deutschen Universitätsklinika zeigt: Der Frauenanteil in Oberarztpositionen beträgt 45%, in Oberarztpositionen mit Professur 31%, und in leitenden Oberarztpositionen mit Professur lediglich 18% 5 .

Besonders ausgeprägt sind die geschlechterspezifischen Unterschiede in chirurgischen Fächern sowie in der Gastroenterologie. So lag der Anteil weiblicher Fachärztinnen für Gastroenterologie und Hepatologie in Österreich im Jahr 2020 bei 55%, jedoch waren nur 17% der Spitzenpositionen in diesem Fachgebiet mit Frauen besetzt 6 . Innerhalb der gastrointestinalen Endoskopie ist diese Diskrepanz besonders evident. Die Ursachen sind vielfältig: Sie reichen von geschlechterspezifischen Vorurteilen, tradierten Rollenbildern und Stereotypen über Bedenken bezüglich Strahlenexposition und Schwangerschaft bis hin zur horizontalen und vertikalen Segregation von Frauen in medizinischen Fachgebieten. Auch der Mangel an weiblichen Vorbildern und Mentoren trägt wesentlich dazu bei, dass Frauen Führungspositionen in diesen traditionell männlich dominierten Bereichen seltener anstreben. Dieser Mangel wirkt sich direkt auf die nachfolgenden Generationen aus: Frauen streben seltener endoskopisch-interventionelle Spezialisierungen an, wenn es an identifikationsstiftenden Vorbildern fehlt.

Das Bewusstsein über diese strukturellen Ungleichheiten ist ein entscheidender Schritt, um die Mechanismen geschlechterspezifischer Benachteiligung zu analysieren und zielgerichtete Maßnahmen zu entwickeln. Nur durch eine systematische Auseinandersetzung mit den zugrunde liegenden Barrieren können effektive Gleichstellungsstrategien formuliert und nachhaltig umgesetzt werden.

Vor diesem Hintergrund wurde durch die Initiative der „Endoskopikerinnen“ eine anonyme Online-Umfrage unter Gastroenterologinnen und Gastroenterologen in Deutschland initiiert. Ziel war es, den aktuellen Stand geschlechterspezifischer Unterschiede in der endoskopischen Tätigkeit sowie die subjektiv wahrgenommenen Barrieren und Herausforderungen systematisch zu erfassen.

## 2. Material und Methodik

### 2.1 Online-Umfrage

Im Februar 2025 wurden alle Mitglieder der Deutschen Gesellschaft für Gastroenterologie, Verdauungs- und Stoffwechselkrankheiten (DGVS) eingeladen, an einer anonymen Online-Umfrage teilzunehmen. Ziel der Befragung war es, anhand 16 standardisierter Fragen Informationen zum beruflichen Tätigkeitsfeld, dem Ausbildungsstand, Umfang und Status der endoskopischen Tätigkeit sowie zur Zufriedenheit der fachlichen Weiterbildung zu erheben mit Fokus auf geschlechterspezifische Unterschiede.

### 2.2 Statistische Analyse

Kategoriale Variablen wurden als Prozentangaben dargestellt. Zum Vergleich kategorialer Variablen wurde der χ² (Chi-Quadrat)-Test bzw. Fisher-Exact-Test verwendet. Zur Untersuchung geschlechts-, alters- und arbeitsbezogener Unterschiede in beruflicher Position, Arbeitszeitmodell und Durchführung komplexer endoskopischer Tätigkeiten wurden drei separate multivariate binäre logistische Regressionsmodelle berechnet. Die abhängigen Variablen waren: (1) Führungsposition (Chefarzt*in = 1 vs. alle anderen Positionen = 0), (2) Teilzeittätigkeit (ja = 1 vs. nein = 0) sowie (3) Durchführung komplexer endoskopischer Tätigkeiten (ja = 1 vs. nein = 0). In allen Modellen wurden Geschlecht (männlich vs. weiblich), Alter (<30, 30–39, 40–49, 50–59, ≥60 Jahre) sowie der Arbeitsort (Grund- und Regelversorgung, Maximalversorger, Universitätsklinikum, Privatklinik, Praxis bzw. Praxis mit stationären Belegbetten) als Kovariablen berücksichtigt. Abhängig von der Fragestellung wurde zusätzlich entweder die berufliche Position oder der Beschäftigungsumfang (Vollzeit vs. Teilzeit) in die Modelle aufgenommen. Referenzkategorien wurden a priori definiert (weiblich, altersabhängig jeweils die größte bzw. inhaltlich zentrale Gruppe, Fachärzt*in, Vollzeit, Grund- und Regelversorgung). Die Ergebnisse werden als Odds Ratios (OR) mit 95%-Konfidenzintervallen (KI) angegeben. Die Modellgüte wurde anhand des McFadden-Pseudo-R² bewertet. Ein Signifikanzniveau von p<0,05 wurde als statistisch signifikant definiert. Die statistischen Analysen erfolgten mit der Software R, Version 4.2.3 und jamovi, Version 2.6.

## 3. Ergebnisse

### 3.1 Demografische Merkmale

**Abb. 1:**
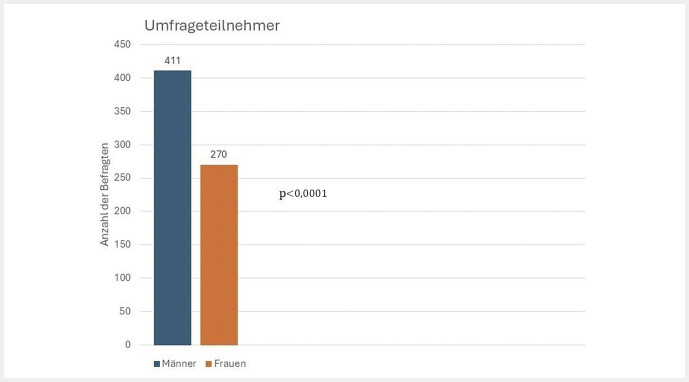
Geschlechtsspezifische Verteilung der Befragten.

Von insgesamt 7362 DGVS Mitgliedern nahmen 685 Personen (9,30%) an der Umfrage teil. Davon waren 270 (39,6%) weiblich und 411 (60,4%) männlich (p<0,0001) ( Abb. 1 ). Die demografischen Merkmale der Teilnehmenden sind in Tab. 1 dargestellt. Der überwiegende Teil der Befragten befand sich im Alter zwischen 40 und 59 Jahren (32,31%). In den Altersgruppen 50–59 Jahre sowie ≥60 Jahre war der Anteil männlicher Befragter höher als der weibliche (31,46% versus 25,93%, p=0,1424 und 22,44% versus 6,30%, p<0,0001). Umgekehrt zeigte sich in der Altersgruppe 30–39 Jahre sowie 40–49 Jahre ein höherer Anteil weiblicher Teilnehmender (15,85% versus 30,74%, p<0,0001 und 29,51% versus 36,67%, p=0,0618). Eine Subgruppenanalyse nach Versorgungsstruktur (Universitätskliniken/Maximalversorger vs. Grund- und Regelversorger/Privatkliniken vs. Praxen) ergab ein vergleichbares Muster der geschlechtsspezifischen Altersverteilung ( Tab. 2 ).

**Table:** **Tab. 1** Geschlechtsspezifischer Vergleich der Ergebnisse der anonymen Online-Umfrage basierend auf 16 standardisierten Fragen.

	Gesamt n (%)	Unbeantwortet, n (%)	Männlich, n (%)	Weiblich, n (%)	P-Wert
	**685 (100%)**				
**Geschlecht (↔)**	**681 (100%)**	**4**	**411 (60,35%)**	**270 (39,65%)**	**<0,0001**
**Alter (↕)**	**684 (100%)**	**1**	**410 (100%)**	**270 (100%)**	**<0,0001**
<30 Jahre	4 (0,58%)		3 (0,73%)	1 (0,37%)	1,0000*
30–39 Jahre	148 (21,64%)		65 (15,85%)	83 (30,74%)	<0,0001
40–49 Jahre	221 (32,31%)		121 (29,51%)	99 (36,67%)	0,0618
50–59 Jahre	199 (29,09%)		129 (31,46%)	70 (25,93%)	0,1424
>60 Jahre	112 (16,37%)		92 (22,44%)	17 (6,30%)	<0,0001
**Facharztbezeichnung (↕)**	**684 (100%)**	**1**	**411 (100%)**	**269 (100%)**	**0,5393**
In Weiterbildung zur Fachärzt*in für Innere Medizin	13 (1,90%)		7 (1,70%)	6 (2,23%)	0,8378
Fachärzt*in für Innere Medizin	76 (11,11%)		41 (9,98%)	35 (13,01%)	0,2696
In Weiterbildung zur Fachärzt*in für Gastroenterologie	58 (8,48%)		32 (7,79%)	26 (9,67%)	0,4730
Fachärzt*in für Gastroenterologie	533 (77,92%)		329 (80,05%)	200 (74,35%)	0,0981
In Weiterbildung zur Fachärzt*in für Allgemeinchirurgie	0 (0%)		0 (0%)	0 (0%)	1,0000*
Fachärzt*in für Allgemeinchirurgie	4 (0,58%)		2 (0,49%)	2 (0,74%)	0,6501*
**Arbeitsort (↕)**	**681 (100%)**	**4**	**409 (100%)**	**268 (100%)**	**0,3533**
Universitätsklinikum	82 (12,04%)		43 (10,51%)	39 (14,55%)	0,1458
Maximalversorger	116 (17,03%)		68 (16,63%)	47 (17,54%)	0,8382
Grund- und Regelversorger	248 (36,42%)		145 (35,45%)	102 (38,06%)	0,5435
Privatklinik	5 (0.73%)		3 (0,73%)	2 (0,75%)	1,0000*
Praxis mit stationären Belegbetten	7 (1,03%)		4 (0,98%)	3 (1,12%)	1,0000*
Praxis	223 (32,75%)		146 (35,70%)	75 (27,99%)	0,0446
**Position (↕)**	**684 (100%)**	**1**	**411 (100%)**	**269 (100%)**	**<0,0001**
Chefärzt*in	78 (11,40%)		63 (15,33%)	15 (5,58%)	0,0002
Ltd Ärzt*in	84 (12,28%)		54 (13,14%)	30 (11,15%)	0,5153
Oberärzt*in	237 (34,65%)		126 (30,66%)	108 (40,15%)	0,0137
Fachärzt*in	79 (11,55%)		33 (8,03%)	46 (17,10%)	0,0005
Assistenzärzt*in	28 (4,09%)		13 (3,16%)	15 (5,58%)	0,1766
Fachärzt*in in Niederlassung	178 (26,02%)		122 (29,68%)	55 (20,45%)	0,0095
Studierende	0 (0%)		0 (0%)	0 (0%)	1,0000*
**Arbeitszeitmodell (↕)**	**684 (100%)**	**1**	**411 (100%)**	**269 (100%)**	**<0,0001**
Vollzeit	503 (73,54%)		348 (84,67%)	153 (56,88%)	<0,0001
Teilzeit	181 (26,46%)		63 (15,33%)	116 (43,12%)	<0,0001
Schichtarbeit	0 (0%)		0 (0%)	0 (0%)	1,0000*
**Endoskopische Tätigkeit (↕)**	**681 (100%)**	**4**	**410 (100%)**	**268 (100%)**	1,0000*
Ja	679 (99,71%)		409 (99,76%)	267 (99,63%)	1,0000*
Nein	2 (0,29%)		1 (0,24%)	1 (0,37%)	1,0000*
**Dauer der endoskopischen Tätigkeit (↕)**	**684 (100%)**	**1**	**410 (100%)**	**270 (100%)**	**<0,0001**
<2 Jahre	28 (4,09%)		14 (3,41%)	14 (5,19%)	0,3474
2–5 Jahre	100 (14,62%)		38 (9,27%)	62 (22,96%)	<0,0001
5–10 Jahre	104 (15,20%)		47 (11,46%)	56 (20,74%)	0,0014
>10 Jahre	452 (66,08%)		311 (75,85%)	138 (51,11%)	<0,0001
**Art der endoskopischen Untersuchungen, die routinemäßig durchgeführt werden (Mehrfachauswahl) (↕)**	**684 (100%)**	**1**	**410 (100%)**	**270 (100%)**	
Diagnostische ÖGD	676 (98,83%)		406 (99,02%)	266 (98,52%)	0,7191*
Diagnostische Koloskopie	667 (97,51%)		402 (98,05%)	261 (96,67%)	0,3797
Koloskopie mit Schlingen-Polypektomie bis 2 cm Größe	655 (95,76%)		399 (97,32%)	252 (93,33%)	0,0203
Endoskopische Blutstillung	602 (88,01%)		377 (91,95%)	221 (81,85%)	0,0001
Komplexe Polypenentfernung unterer GI-Trakt: EMRPolypen >2 cm Größe, eFTR	422 (61,70%)		290 (70,73%)	130 (48,15%)	<0,0001
Komplexe Läsionsentfernung oberer GI-Trakt: EMRBarrettläsionen, Duodenaladenome, eFTR	238 (34,80%)		177 (43,17%)	61 (22,59%)	<0,0001
ESD oberer oder unterer GI-Trakt	77 (11,26%)		64 (15,61%)	13 (4,81%)	<0,0001
POEM und/oder G-POEM und/oder Z-POEM	26 (3,80%)		20 (4,88%)	6 (2,22%)	0,1181
Zenkerdivertikulotomie	132 (19,30%)		100 (24,39%)	32 (11,85%)	0,0001
Single- oder Doppleballonenteroskopie	193 (28,22%)		140 (34,15%)	53 (19,63%)	0,0001
Diagnostische EUS	346 (50,58%)		219 (53,41%)	127 (47,00%)	0,1213
Diagnostische EUS mit Punktion	276 (40,35%)		190 (46,34%)	86 (31,85%)	0,0002
Therapeutische EUS inklusive Drainageverfahren Pankreaszysten/Nekrosektomie	187 (27,34%)		140 (34,14%)	47 (17,40%)	<0,0001
Therapeutische EUS inklusive Rendezvous Verfahren, EUS- Anastomosen (CDS/HGS/GE)	97 (14,18%)		79 (19,27%)	18 (6,67%)	<0,0001
ERCP mit EPT, einfache Steinextraktion, einfache Stentwechsel	357 (52,19%)		229 (55,85%)	128 (47,40%)	0,0376
Komplexe ERCP mit Stenting komplexer GG-Stenosen (Bismuth IV-Situation, PSC), Pankreasgangintervention, Cholangioskopie (diagnostisch/therapeutisch), postoperative Anatomie	242 (35,38%)		172 (41,95%)	70 (25.93%)	<0,0001
Bariatrische Eingriffe, z.B. ESG, Outletrepair	18 (2,63%)		15 (3,65%)	3 (1,11%)	0,0506*
**Zufriedenheit des aktuellen Standes der endoskopischen Tätigkeit/Ausbildung (↕)**	**683 (100%)**	**2**	**411 (100%)**	**269 (100%)**	**<0,0001**
Ja	481 (70,42%)		325 (79,08%)	155 (57,62%)	<0,0001
Nein	202 (29,58%)		86 (20,92%)	114 (42,38%)	<0,0001
**Ursache für Unzufriedenheit (Mehrfachauswahl möglich) (↕)**	**210 (100%)**	**475**	**92 (100%)**	**116 (100%)**	
Noch nicht so weit in der Ausbildung/Reihenfolge	63 (30,0%)		28 (30,43%)	34 (29,31%)	0,9813
Kind/Familie	63 (30,0%)		15 (16,30%)	48 (41,38%)	0,0002
Teilzeittätigkeit	57 (27,14%)		10 (10,87%)	46 (39,66%)	<0,0001
Sonstiges	119 (56,67%)		55 (59,78%)	64 (55,17%)	0,5986
**Wenn unzufrieden, in welchem Bereich ist verstärkt Weiterbildung gewünscht (↕)**	**293 (100%)**	**392**	**146 (100%)**	**145 (100%)**	**0,0003**
Komplexe Resektionstechniken (EMR großer Läsionen, eFTR, ESD)	82 (27,99%)		43 (29,45%)	39 (26,90%)	0,7232
Tunneltechniken (POEM, G-POEM, Z-POEM)	33 (11,26%)		28 (19,18%)	5 (3,45%)	0,0001
Therapeutische ERCP	60 (20,48%)		24 (16,44%)	35 (24,14%)	0,1368
Therapeutische EUS	81 (27,65%)		32 (21,92%)	48 (33,10%)	0,0449
Anderes	37 (12,63%)		19 (13,01%)	18 (12,41%)	1,0000
**Weitere Tätigkeit neben der klinischen Tätigkeit (keine Mehrfachauswahl) (↕)**	**669 (100%)**	**16**	**404 (100%)**	**262 (100%)**	**<0,0001**
Ja als Referent*in im Rahmen von regionalen Fortbildungsveranstaltungen	144 (21,52%)		102 (25,25%)	41 (15,65%)	0,0044
Ja als Referent*in im Rahmen von überregionalen Fortbildungsveranstaltungen und Tagungen der Fachgesellschaften	75 (11,21%)		52 (12,87%)	23 (8,78%)	0,1319
Ja als Referent*in/Tutor*in im Rahmen von endoskopischen Trainingskursen	50 (7,47%)		36 (8,91%)	14 (5,34%)	0,1197
Ja als Live Demonstrierende/Demonstrierender im Rahmen von Live-Endoskopieveranstaltungen	19 (2,84%)		16 (3,96%)	3 (1,15%)	0,0336*
Nein	381 (56,95%)		198 (49,01%)	181 (69,08%)	<0,0001
**Gründe, warum nicht (↕)**	**400 (100%)**	**285**	**207 (100%)**	**191 (100%)**	**0,0004**
Noch nicht so weit in der Aus-/Weiterbildung	46 (11,50%)		21 (10,14%)	25 (13,09%)	0,4467
Zeit für Familie/Privates/Freizeit ist mir wichtiger	148 (37,00%)		85 (41,06%)	62 (32,46%)	0,0944
Traue ich mir nicht zu/Habe ich mir nicht zugetraut	78 (19,50%)		24 (11,59%)	54 (28,27%)	<0,0001
Hatte nie die Gelegenheit, würde mich aber gerne Einbringen	70 (17,50%)		41 (19,81%)	28 (14,66%)	0,2215
Anderes	58 (14,50%)		36 (17,39%)	22 (11,52%)	0,1293
**Wissenschaftliche Arbeit im Bereich der endoskopischen Forschung (↕)**	**680 (100%)**	**5**	**408 (100%)**	**269 (100%)**	**0,0186**
Ja	84 (12,35%)		61 (14,95%)	23 (8,55%)	0,0186
Nein	596 (87,65%)		347 (85,05%)	246 (91,45%)	0,0186
**Zukünftiger Tätigkeitsbereichswunsch (↕)**	**675 (100%)**	**10**	**406 (100%)**	**266 (100%)**	**0,1681**
Status idem	434 (64,30%)		268 (66,01%)	165 (62,03%)	0,3313
Universitätsklinikum	31 (4,59%)		11 (2,71%)	20 (7,52%)	0,0066
Maximalversorger	56 (8,30%)		32 (7,88%)	23 (8,62%)	0,8338
Grund- und Regelversorger	34 (5,04%)		22 (5,42%)	12 (4,51%)	0,7302
Privatklinik	5 (0,74%)		3 (0,74%)	2 (0,75%)	1,0000*
Praxis mit stationären Belegbetten	26 (3,85%)		17 (4,19%)	9 (3,38%)	0,7461
Praxis	89 (13,19%)		53 (13,05%)	35 (13,16%)	1,0000
(↔): prozentuale Verteilung/Berechnung innerhalb der Zeilen (↕): prozentuale Verteilung/Berechnung innerhalb der Spalten Chi-Quadrat (X ^2^ )-Test * Fischer-Exakt-Test					

**Table:** **Tab. 2** Vergleich der Geschlechterverteilung nach Versorgungsstruktur (Universitätsklinikum/Maximalversorger versus Grund- und Regelversorger/Privatklinik versus Praxis mit stationären Betten/Praxis).

	Universitätsklinikum und Maximalversorger				Grund- und Regelversorger, Privatklinik				Praxis mit stationären Betten, Praxis			
	Gesamt, n (%)	Männlich, n (%)	Weiblich, n (%)	P-Wert	Gesamt, n (%)	Männlich, n (%)	Weiblich, n (%)	P-Wert	Gesamt, n (%)	Männlich, n (%)	Weiblich, n (%)	P-Wert
	**198 (100%)**				**253 (100%)**				**230 (100%)**			
**Geschlecht (↔)**	**197 (100%)**	**111 (56,35%)**	**86 (43,65%)**	**0,0749**	**252 (100%)**	**148 (58,73%)**	**104 (41,27%)**	**0,0056**	**228 (100%)**	**150 (65,79%)**	**78 (34,21%)**	**0,0001**
**Alter (↕)**	**198 (100%)**	**111 (100%)**	**86 (100%)**	**0,0019**	**253 (100%)**	**148 (100%)**	**104 (100%)**	**0,0038**	**229 (100%)**	**149 (100%)**	**78 (100%)**	**0,0004**
<30 Jahre	3 (1,52%)	3 (2,70%)	0 (0%)	0,2583*	1 (0,40%)	0 (0%)	1 (0,96%)	0,4127*	0 (0%)	0 (0%)	0 (0%)	1,0000*
30–39 Jahre	74 (37,37%)	30 (27,03%)	44 (51,16%)	0,0009	64 (25,30%)	32 (21,62%)	32 (30,77%)	0,1348	10 (4,37%)	3 (2,01%)	7 (8,97%)	0,0343*
40–49 Jahre	65 (32,82%)	39 (35,14%)	26 (30,23%)	0,5666	89 (35,18%)	46 (31,08%)	43 (41,35%)	0,1224	65 (28,38%)	36 (24,16%)	29 (37,18%)	0,0566
50–59 Jahre	40 (20,20%)	26 (23,42%)	14 (16,30%)	0,2902	69 (27,27%)	45 (30,41%)	24 (23,08%)	0,2539	89 (38,86%)	57 (38.26%)	32 (41,03%)	0,7926
> 60 Jahre	15 (7,58%)	13 (11,71%)	2 (2.33%)	0.0146*	29 (11,46%)	25 (16,89%)	4 (3,85%)	0,0011*	65 (28,38%)	53 (35,57%)	10 (12,82%)	0,0005
**Facharztbezeichnung (↕)**	**198 (100%)**	**111 (100%)**	**86 (100%)**	**0,5502**	**253 (100%)**	**148 (100%)**	**104 (100%)**	**0,6626**	**230 (100%)**	**150 (100%)**	**78 (100%)**	**0,6345**
In Weiterbildung zur Fachärzt*in für Innere Medizin	4 (2,02%)	3 (2,70%)	1 (1.16%)	0,6334*	9 (3,56%)	4 (2,70%)	5 (4,81%)	0,4946*	0 (0%)	0 (0%)	0 (0%)	1,0000*
Fachärzt*in für Innere Medizin	30 (15,15%)	13 (11,71%)	17 (19,76%)	0,1736	30 (11,86%)	16 (10,81%)	14 (13,46%)	0,6584	16 (6,96%)	12 (8,0%)	4 (5,13%)	0,5867*
In Weiterbildung zur Fachärzt*in für Gastroenterologie	34 (17,17%)	19 (17,12%)	15 (17,44%)	1,0000	15 (5,93%)	8 (5,41%)	7 (6,73%)	0,8671	8 (3,48%)	4 (2,67%)	4 (5,13%)	0,4503*
Fachärzt*in für Gastroenterologie	127 (64,14%)	75 (67,57%)	52 (60,47%)	0,3773	199 (78,66%)	120 (81,08%)	78 (75,0%)	0,3162	204 (88,70%)	133 (88,66%)	69 (88,46%)	1,0000
In Weiterbildung zur Fachärzt*in für Allgemeinchirurgie	0 (0%)	0 (0%)	0 (0%)	1,0000*	0 (0%)	0 (0%)	0 (0%)	1,0000*	0 (0%)	0 (0%)	0 (0%)	1,0000*
Fachärzt*in für Allgemeinchirurgie	2 (1,01%)	1 (0,91%)	1 (1,16%)	1,0000*	0 (0%)	0 (0%)	0 (0%)	1,0000*	2 (0.87%)	1 (0,67%)	1 (1,28%)	1,0000*
**Position (↕)**	**198 (100%)**	**111 (100%)**	**86 (100%)**	**0,0027**	**253 (100%)**	**148 (100%)**	**104 (100%)**	**0,0005**	**230 (100%)**	**150 (100%)**	**78 (100%)**	**0,0863**
Chefärzt*in	18 (9,09%)	16 (14,41%)	2 (2,33%)	0,0048*	59 (23,32%)	47 (31,76%)	12 (11,54%)	0,0003	1 (0,43%)	0 (0%)	1 (1,28%)	0,3421*
Ltd Ärzt*in	30 (15,15%)	16 (14,41%)	14 (16,28%)	0,8718	39 (15,42%)	27 (18,24%)	12 (11,54%)	0,2034	13 (5,65%)	9 (6,0%)	4 (5,13%)	1,0000*
Oberärzt*in	102 (51,52%)	61 (54,95%)	40 (46,51%)	0,3020	131 (51,78%)	62 (41,90%)	68 (65,38%)	0,0004	3 (1,30%)	3 (2,0%)	0 (0%)	0,5529*
Fachärzt*in	33 (16,67%)	11 (9,91%)	22 (25,58%)	0,0064	13 (5,14%)	7 (4,73%)	6 (5,77%)	0,9378	32 (13,91%)	15 (10,0%)	17 (21.80%)	0,0256
Assistenzärzt*in	15 (7,58%)	7 (6,31%)	8 (9,30%)	0,6062	11 (4,35%)	5 (3,38%)	6 (5.77%)	0,3689*	2 (0,87%)	1 (0,67%)	1 (1.28%)	1.0000*
Fachärzt*in in Niederlassung	0 (0%)	0 (0%)	0 (0%)	1,0000*	0 (0%)	0 (0%)	0 (0%)	1,0000*	178 (77,39%)	122 (81,33%)	55 (70.51%)	0.0905
Studierende	0 (0%)	0 (0%)	0 (0%)	1,0000*	0 (0%)	0 (0%)	0 (0%)	1,0000*	0 (0%)	0 (0%)	0 (0%)	1.0000*
**Arbeitszeitmodell (↕)**	**198 (100%)**	**111 (100%)**	**86 (100%)**	**0,0001**	**253 (100%)**	**148 (100%)**	**104 (100%)**	**<0,0001**	**230 (100%)**	**150 (100%)**	**78 (100%)**	**<0,0001**
Vollzeit	154 (77,78%)	98 (88,29%)	55 (63,95%)	0,0001	181 (71,54%)	122 (82,43%)	59 (56,73%)	<0,0001	165 (71,74%)	126 (84,0%)	38 (48.72%)	0.0000
Teilzeit	44 (22,22%)	13 (11,71%)	31 (36,04%)	0,0001	72 (28,46%)	26 (17,57%)	45 (43,27%)	<0,0001	65 (28,26%)	24 (16,0%)	40 (51,28%)	<0,0001
Schichtarbeit	0 (0%)	0 (0%)	0 (0%)	1,0000*	0 (0%)	0 (0%)	0 (0%)	1,0000*	0 (0%)	0 (0%)	0 (0%)	1,0000*
**Endoskopische Tätigkeit (↕)**	**197 (100%)**	**111 (100%)**	**85 (100%)**	**1,0000**	**251 (100%)**	**147 (100%)**	**104 (100%)**	**0,0066**	**230 (100%)**	**150 (100%)**	**78 (100%)**	**<0,0001**
Ja	194 (98,48%)	110 (99,10%)	84 (98,82%)	1,0000	251 (100%)	147 (100%)	104 (100%)	1,0000*	230 (100%)	150 (100%)	78 (100%)	1,0000*
Nein	2 (1,02%)	1 (0,90%)	1 (1,18%)	1,0000*	0 (0%)	0 (0%)	0 (0%)	1,0000*	0 (0%)	0 (0%)	0 (0%)	1,0000*
**Dauer der endoskopischen Tätigkeit (↕)**	**197 (100%)**	**110 (100%)**	**86 (100%)**	**0,0036**	**253 (100%)**	**148 (100%)**	**104 (100%)**	**0,0001**	**230 (100%)**	**150 (100%)**	**78 (100%)**	**0,0090**
<2 Jahre	16 (8,12%)	6 (5,45%)	10 (11,63%)	0,1924	11 (4,35%)	7 (4,73%)	4 (3,85%)	1,0000*	1 (0,43%)	1 (0,67%)	0 (0%)	1,0000*
2–5 Jahre	47 (23,86%)	19 (17,27%)	28 (32,56%)	0,0204	45 (17,79%)	18 (12,16%)	27 (25,96%)	0,0081	7 (3,04%)	1 (0,67%)	6 (7,69%)	0,0071*
5–10 Jahre	37 (18,78%)	19 (17,27%)	18 (20,93%)	0,6416	48 (18,97%)	19 (12,84%)	29 (27,88%)	0,0046	18 (7,83%)	9 (6,0%)	9 (11,54%)	0,2253
>10 Jahre	96 (48,73%)	66 (60,0%)	30 (34,88%)	0,0008	149 (58,89%)	104 (70,27%)	44 (42,31%)	<0,0001	204 (88,70%)	139 (92,67%)	63 (80,77%)	0,0138
**Art der endoskopischen Untersuchungen, die routinemäßig durchgeführt werden (Mehrfachauswahl) (↕)**	**198 (100%)**	**111 (100%)**	**86 (100%)**		**253 (100%)**	**148 (100%)**	**104 (100%)**		**230 (100%)**	**150 (100%)**	**78 (100%)**	
Diagnostische ÖGD	194 (97,98%)	109 (98,20%)	85 (98,84%)	1,0000*	252 (99,60%)	147 (99,32%)	104 (100%)	1,0000*	227 (98,70%)	148 (98,67%)	77 (98,72%)	1,0000*
Diagnostische Koloskopie	189 (95,45%)	107 (96,40%)	82 (95,35%)	0,7308*	248 (98,02%)	144 (97,30%)	103 (99,04%)	0,6516*	226 (98,26%)	149 (99,33%)	75 (96,15%)	0,1171*
Koloskopie mit Schlingen-Polypektomie bis 2cm Größe	186 (93,94%)	107 (96,40%)	79 (91,86%)	0,2158*	242 (95,65%)	143 (96,62%)	98 (94,23%)	0,3689*	225 (97,83%)	148 (98,67%)	75 (96,15%)	0,3413*
Endoskopische Blutstillung	185 (93,43%)	106 (95,50%)	79 (91,86%)	0,4487	240 (94,86%)	143 (96,62%)	96 (92,31%)	0,2168	175 (76,09%)	126 (84,0%)	47 (60,26%)	0,0001
Komplexe Polypenentfernung unterer GI-Trakt: EMR Polypen >2 cm Größe, eFTR	134 (67,68%)	86 (77,48%)	48 (55,81%)	0,0021	186 (73,52%)	122 (82,43%)	63 (60,58%)	0,0002	100 (43,48%)	80 (53,33%)	19 (24,36%)	0,0001
Komplexe Läsionsentfernung oberer GI-Trakt: EMR Barrettläsionen, Duodenaladenome, eFTR	98 (49,49%)	68 (61,26%)	30 (34,88%)	0,0004	120 (47,43%)	89 (60,14%)	31 (29,81%)	<0,0001	18 (7,83%)	18 (12,0%)	0 (0%)	0,0005*
ESD oberer oder unterer GI Trakt	43 (21,72%)	34 (30,63%)	9 (10,47%)	0,0013	31 (12,25%)	27 (18,24%)	4 (3,85%)	0,0004*	3 (1,30%)	3 (2,0%)	0 (0%)	0,5529*
POEM und/oder G-POEM und/oder Z-POEM	19 (9,60%)	14 (12,61%)	5 (5,81%)	0,1739	6 (2,37%)	5 (3,38%)	1 (0,96%)	0,4054*	1 (0,43%)	1 (0,67%)	0 (0%)	1,0000*
Zenkerdivertikulotomie	61 (30,81%)	42 (37,84%)	19 (22,09%)	0,0267	68 (26,88%)	55 (37,16%)	13 (12,5%)	<0,0001	3 (1,30%)	3 (2,0%)	0 (0%)	0,5529*
Single- oder Doppleballonenteroskopie	99 (50,0%)	72 (64,86%)	27 (31,40%)	<0,0001	89 (35,18%)	63 (42,47%)	25 (24,04%)	0,0037	4 (1,74%)	4 (2,67%)	0 (0%)	0,3018*
Diagnostische EUS	141 (71,21%)	88 (79,30%)	53 (61,63%)	0,0103	194 (76,68%)	121 (81,76%)	72 (69,23%)	0,0307	11 (4,78%)	10 (6,67%)	1 (1,28%)	1,1030*
Diagnostische EUS mit Punktion	109 (55,10%)	76 (68,47%)	33 (38,37%)	<0,0001	160 (63,24%)	108 (72,97%)	51 (49,04%)	0,0002	7 (3,04%)	6 (4,0%)	1 (1,28%)	0,4270*
Therapeutische EUS inklusive Drainageverfahren Pankreaszysten/Nekrosektomie	85 (42,93%)	62 (55,86%)	23 (26,74%)	0,0001	98 (38,74%)	75 (50,68%)	23 (22,12%)	<0,0001	4 (1,74%)	3 (2,0%)	1 (1,28%)	1,0000*
Therapeutische EUS inklusive Rendezvous Verfahren, EUS- Anastomosen (CDS/HGS/GE)	49 (24,75%)	39 (35,14%)	10 (11,63%)	0,0003	47 (18,58%)	39 (26,35%)	8 (7,70%)	0,0003	1 (0,43%)	1 (0,67%)	0 (0%)	1,0000*
ERCP mit EPT, einfache Steinextraktion, einfache Stentwechsel	143 (72,22%)	90 (81,08%)	53 (61,63%)	0,0040	200 (79,05%)	127 (85,81%)	72 (69,23%)	0,0025	12 (5,22%)	10 (6,67%)	2 (2,56%)	0,2281*
Komplexe ERCP mit Stenting komplexer GG-Stenosen (Bismuth IV Situation, PSC), Pankreasgangintervention, Cholangioskopie (diagnostisch/therapeutisch), postoperative Anatomie	111 (56,06%)	78 (70,27%)	33 (38,37%)	<0,0001	123 (48,62%)	88 (59,46%)	35 (33,65%)	0,0001	6 (2,61%)	4 (2,67%)	2 (2,56%)	1,0000*
Bariatrische Eingriffe, z.B. ESG, Outletrepair	9 (4,55%)	7 (6,31%)	2 (2,33%)	0,3036*	8 (3,16%)	7 (4,73%)	1 (0,96%)	0,1454*	1 (0,43%)	1 (0,67%)	0 (0%)	1,0000*
**Zufriedenheit mit aktuellen Stand der endoskopischen Tätigkeit/Ausbildung (↕)**	**198 (100%)**	**111 (100%)**	**86 (100%)**	**<0,0001**	**253 (100%)**	**148 (100%)**	**104 (100%)**	**0,0051**	**229 (100%)**	**150 (100%)**	**78 (100%)**	**0,0256**
Ja	121 (61,11%)	83 (74,77%)	38 (44,19%)	<0,0001	160 (63,24%)	105 (70,95%)	55 (52,88%)	0,0051	197 (86,03%)	135 (90,0%)	61 (78,21%)	0,0256
Nein	77 (38,89%)	28 (25,23%)	48 (55,81%)	<0,0001	93 (36,76%)	43 (29,05%)	49 (47,12%)	0,0051	32 (13,97%)	15 (10,0%)	17 (21,79%)	0,0256
**Ursache für Unzufriedenheit (Mehrfachauswahl möglich) (↕)**	**78 (100%)**	**28 (100%)**	**49 (100%)**		**96 (100%)**	**45 (100%)**	**50 (100%)**		**36 (100%)**	**19 (100%)**	**17 (100%)**	
Noch nicht so weit in der Ausbildung/Reihenfolge	32 (41,03%)	13 (46,43%)	19 (38,77%)	0,6780	28 (29,17%)	14 (31,11%)	14 (28,0%)	0,9150	3 (8,33%)	2 (10,53%)	1 (5,88%)	1,0000*
Kind/Familie	24 (30,77%)	4 (14,29%)	20 (40,82%)	0,0210*	29 (30,21%)	9 (20,0%)	20 (40,0%)	0,0587	10 (27,78%)	2 (10,53%)	8 (47,06%)	0,0248*
Teilzeittätigkeit	16 (20,51%)	1 (3,57%)	15 (30,61%)	0,0070*	24 (25,0%)	5 (11,11%)	19 (38,0%)	0,0055	17 (47,22%)	5 (26,32%)	12 (70,59%)	0,0202
Sonstiges	42 (53,85%)	15 (53,57%)	27 (55,10%)	1,0000	53 (55,21%)	28 (62,22%)	25 (50,0%)	0,3218	24 (66,67%)	12 (63,16%)	12 (70,59%)	0,9060
**Wenn unzufrieden, in welchem Bereich ist verstärkt Weiterbildung gewünscht (↕)**	**111 (100%)**	**49 (100%)**	**61 (100%)**	**0,1144**	**143 (100%)**	**75 (100%)**	**68 (100%)**		**39 (100%)**	**22 (100%)**	**17 (100%)**	**0.2376**
Komplexe Resektionstechniken (EMR großer Läsionen, eFTR, ESD)	24 (21,62%)	12 (24,49%)	12 (19,67%)	0,7071	35 (24,48%)	18 (24,0%)	17 (25,37%)	1,0000	23 (58,97%)	13 (59,09%)	10 (58,82%)	1,0000
Tunneltechniken (POEM, G-POEM, Z-POEM)	16 (14,41%)	11 (22,45%)	5 (8,20%)	0,0665	17 (11,89%)	17 (22,67%)	0 (0%)	<0,0001*	0 (0%)	0 (0%)	0 (0%)	1,0000*
Therapeutische ERCP	30 (27,03%)	10 (20,41%)	20 (32,79%)	0,2174	30 (20,98%)	14 (18,67%)	16 (23,88%)	0,5796	0 (0%)	0 (0%)	0 (0%)	1,0000*
Therapeutische EUS	28 (25,23%)	9 (18,37%)	18 (29,51%)	0,2599	50 (34,97%)	20 (26,67%)	30 (44,78%)	0,0376	3 (7,69%)	3 (13,64%)	0 (0%)	0,2429*
Anderes	13 (11,71%)	7 (14,29%)	6 (9,84%)	0,6735	11 (7,69%)	6 (8,0%)	5 (7,46%)	1,0000	13 (33,33%)	6 (27,27%)	7 (41,18%)	0,5681
**Weitere Tätigkeit neben der klinischen Tätigkeit (keine Mehrfachauswahl) (↕)**	**196 (100%)**	**111 (100%)**	**84 (100%)**	**0,0676**	**243 (100%)**	**143 (100%)**	**100 (100%)**		**227 (100%)**	**149 (100%)**	**77 (100%)**	**0,0671**
Ja als Referent*in im Rahmen von regionalen Fortbildungsveranstaltungen	35 (17,86%)	20 (18,02%)	15 (17,86%)	1,0000	51 (20,99%)	39 (27,46%)	12 (12,0%)	0,0061	57 (25,11%)	43 (28,86%)	14 (18,18%)	0,1118
Ja als Referent*in im Rahmen von überregionalen Fortbildungsveranstaltungen und Tagungen der Fachgesellschaften	37 (18,88%)	24 (21,62%)	13 (15,48%)	0,3685	14 (5,76%)	11 (7,75%)	3 (3,0%)	0,1638*	24 (10,57%)	17 (11,41%)	7 (9,09%)	0,7578
Ja als Referent*in/Tutor*in im Rahmen von endoskopischen Trainingskursen	28 (14,29%)	20 (18,02%)	8 (9,52%)	0,1419	18 (7,41%)	12 (8,45%)	6 (6,0%)	0,6407	4 (1,76%)	4 (2,68%)	0 (0%)	0,3021*
Ja als Live Demonstrierende/Demonstrierender im Rahmen von Live-Endoskopieveranstaltungen	9 (4,59%)	7 (6,30%)	2 (2,38%)	0,3044*	7 (2,88%)	6 (4,23%)	1 (1,0%)	0,2446*	3 (1,32%)	3 (2,01%)	0 (0%)	0,5528*
Nein	87 (44,39%)	40 (36,04%)	46 (54,76%)	0,0138	153 (62,96%)	75 (52,82%)	78 (78,0%)	0,0001	139 (61,23%)	82 (55,03%)	56 (72,73%)	0,0146
**Gründe, warum nicht (↕)**	**97 (100%)**	**43 (100%)**	**53 (100%)**		**160 (100%)**	**79 (100%)**	**81 (100%)**	**0,0065**	**141 (100%)**	**84 (100%)**	**56 (100%)**	**0,0250**
Noch nicht so weit in der Aus-/Weiterbildung	24 (24,74%)	10 (23,26%)	14 (26,42%)	0,9057	19 (11,88%)	10 (12,66%)	9 (11,11%)	0,9537	3 (2,13%)	1 (1,19%)	2 (3,57%)	0,5638*
Zeit für Familie/Privates/Freizeit ist mir wichtiger	21 (21,65%)	14 (32,56%)	7 (13,21%)	0,0421	45 (28,13%)	21 (26,58%)	24 (29,63%)	0,8004	80 (56,74%)	49 (58,33%)	30 (53,57%)	0,7019
Traue ich mir nicht zu/Habe ich mir nicht zugetraut	11 (11,34%)	3 (6,98%)	8 (15,09%)	0,3353*	42 (16,25%)	12 (15,19%)	30 (37,04%)	0,0031	25 (17,73%)	9 (10,71%)	16 (28,57%)	0,0132
Hatte nie die Gelegenheit, würde mich aber gerne einbringen	24 (24,74%)	7 (16,28%)	17 (32,08%)	0,1235	33 (20,63%)	23 (29,11%)	10 (12,35%)	0,0153	13 (9,22%)	11 (13,10%)	2 (3,57%)	0,0754*
Anderes	17 (17.53%)	9 (20,93%)	8 (15,09%)	0,6340	21 (13,13%)	13 (16,46%)	8 (9,88%)	0,3183	20 (,4.18%)	14 (16,67%)	6 (10,71%)	0,4596
**Wissenschaftliche Arbeit im Bereich der endoskopischen Forschung (↕)**	**198 (100%)**	**111 (100%)**	**86 (100%)**	**0,0645**	**251 (100%)**	**146 (100%)**	**104 (100%)**	**0,0239**	**228 (100%)**	**149 (100%)**	**78 (100%)**	**0,3439**
Ja	63 (31,82%)	42 (37,84%)	21 (24,42%)	0,0645	13 (5,18%)	12 (8,22%)	1 (0,96%)	0,0096*	8 (3,51%)	7 (4,70%)	1 (1,28%)	0,2689*
Nein	135 (68,18%)	69 (62,16%)	65 (75,58%)	0,0645	238 (94,82%)	134 (91,78%)	103 (99,04%)	0,0096*	220 (96,49%)	142 (95,30%)	77 (98,72%)	0,2689*
**Zukünftiger Tätigkeitsbereichswunsch (↕)**	**197 (100%)**	**110 (100%)**	**86 (100%)**	**0,0249**	**252 (100%)**	**147 (100%)**	**104 (100%)**	**0.6139**	**222 (100%)**	**146 (100%)**	**75 (100%)**	**0,2690**
Status idem	117 (59,30%)	75 (68,18%)	42 (48,84%)	0,0095	163 (64,68%)	92 (62,59%)	70 (67,31%)	0,5244	150 (67,57%)	98 (67,12%)	52 (69,33%)	0,8563
Universitätsklinikum	26 (13,20%)	8 (7,27%)	18 (20,93%)	0,0097	3 (1,19%)	2 (1,36%)	1 (0,96%)	1,0000*	2 (0,90%)	1 (0,68%)	1 (1,33%)	1,0000*
Maximalversorger	34 (17,26%)	18 (16,36%)	15 (17,44%)	0,9937	21,(8,33%)	13 (.84%)	8 (7,69%)	0,9258	1 (0,45%)	1 (0,68%)	0 (0%)	1,0000*
Grund- und Regelversorger	0 (0%)	0 (0%)	0 (0%)	1,0000*	33 (13,10%)	22 (14,97%)	11 (10,58%)	0,4099	1 (0,45%)	0 (0%)	1 (1,33%)	0,3394*
Privatklinik	0 (0%)	0 (0%)	0 (0%)	1,0000*	4 (1,59%)	3 (2,04%)	1 (0,96%)	0,6440*	1 (0,45%)	0 (0%)	1 (1.33%)	0,3394*
Praxis mit stationären Belegbetten	8 (4,06%)	3 (2,73%)	5 (5,81%)	0,3022*	13 (5,16%)	9 (6,12%)	4 (3,85%)	0,5668*	5 (2,25%)	5 (3,42%)	0 (0%)	0,1693*
Praxis	12 (6,09%)	6 (5,45%)	6 (6,98%)	0,8879	15 (5,95%)	6 (4,08%)	9 (8,65%)	0,2168	62 (27,93%)	41 (28,08%)	20 (26,67%)	0,9490
(↔): prozentuale Verteilung/Berechnung innerhalb der Zeilen (↕): prozentuale Verteilung/Berechnung innerhalb der Spalten Chi-Quadrat (X ^2^ )-Test * Fischer-Exakt-Test												

### 3.2 Berufliche Merkmale und Tätigkeitsbereich

**Abb. 2:**
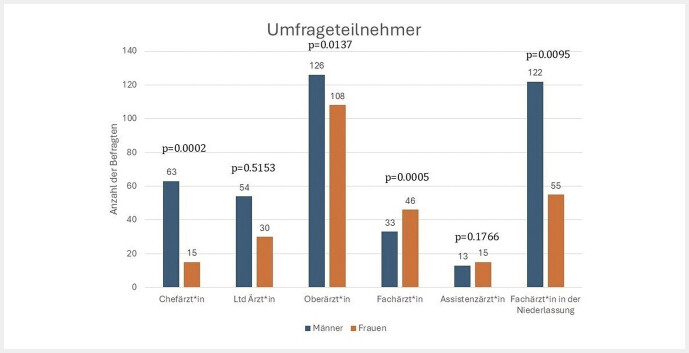
Berufliche Position der Befragten.

**Abb. 3:**
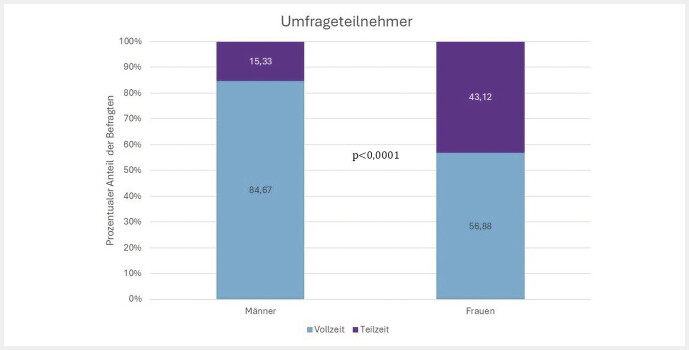
Arbeitszeitmodell der Befragten.

Der überwiegende Teil der Befragten verfügt über die Facharztanerkennung für Gastroenterologie (77,92%). 36,42% der Teilnehmenden waren an Einrichtungen der Grund- und Regelversorgung tätig, gefolgt von 32,75%, die in Praxen arbeiteten. Ein signifikant höherer Anteil männlicher Befragter war in Praxen beschäftigt (35,70% versus 27,99%, p=0,0446). Zudem war ein signifikant höherer Männeranteil als Chefarzt tätig (15,33% versus 5,58%, p=0,0002). Demgegenüber fanden sich signifikant mehr Frauen in den Positionen von Oberärzt:innen und Fachärzt:innen (30,66% versus 40,15%, p=0,0137 und 8,03% versus 17,10%, p=0,0005), ( Abb. 2 ). Die geschlechtsspezifischen Unterschiede innerhalb der hierarchischen Berufsstruktur zeigten sich besonders deutlich auf der Ebene der Chefärzt:innen – unabhängig von der jeweiligen Versorgungsstruktur (Universitätskliniken/Maximalversorger, p=0,0048 vs. Grund- und Regelversorgung/Privatkliniken, p=0,0003), ( Tab. 1 und Tab. 2 ). Darüber hinaus arbeiteten signifikant mehr Frauen in Teilzeit (15,33% versus 43,12%, p<0,0001) ( Abb. 3 ).

### 3.3 Endoskopische Tätigkeit

**Abb. 4:**
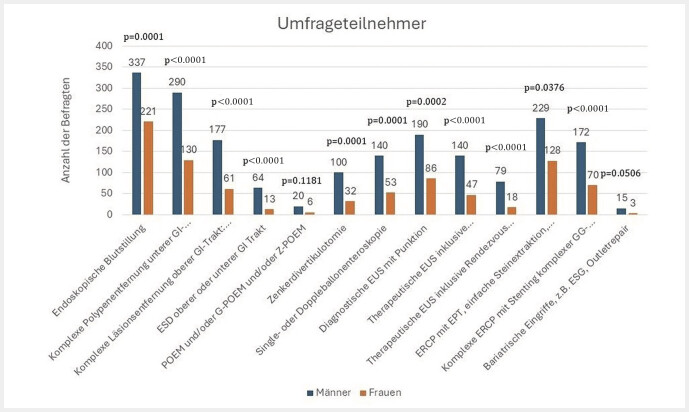
Endoskopische Tätigkeit der Befragten.

Von den befragten Teilnehmenden gaben 99,71% an, endoskopisch tätig zu sein. Der Großteil (66,08%) verfügt über eine endoskopische Erfahrung von mehr als zehn Jahren. Dabei zeigt sich ein signifikanter Geschlechtsunterschied zugunsten der Männer: Sie weisen häufiger eine mehr als zehnjährige endoskopische Erfahrung auf als ihre weiblichen Kolleginnen (75,85% versus 51,11%, p<0,0001) ( Tab. 1 und Abb. 4 ). Bei routinemäßigen diagnostischen Verfahren – wie der diagnostischen Ösophagogastroduodenoskopie (ÖGD) (p=0,7191), Koloskopie (p=0,3797) oder endoskopischen Ultraschalluntersuchung (EUS) (p=0,1213) – zeigten sich keine signifikanten Unterschiede zwischen den Geschlechtern hinsichtlich der Durchführungshäufigkeit. Im Gegensatz dazu traten bei komplexen interventionellen endoskopischen Verfahren – darunter endoskopische Mukosaresektionen (EMR) von Läsionen >2 cm (p<0,0001), endoskopische Submukosadissektionen (ESD) (p<0,0001), Zenkerdivertikulotomien (p=0,0001) therapeutische EUS (p<0,0001) sowie endoskopisch-retrograde Cholangiopankreatikographien (ERCP) mit einfachen Stentwechsel (p=0,0376) sowie komplexe ERCP (p<0,0001) – signifikante Unterschiede auf. Hier lagen die Angaben zur Durchführungshäufigkeit bei Männern zum Teil über 20 Prozentpunkte höher als bei Frauen.

### 3.4 Zufriedenheit mit der aktuellen endoskopischen Tätigkeit und Ausbildung

**Abb. 5:**
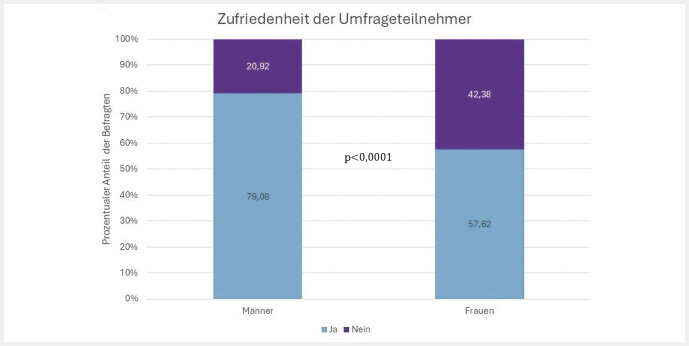
Zufriedenheit der Befragten mit der aktuellen endoskopischen Tätigkeit und Ausbildung.

**Abb. 6:**
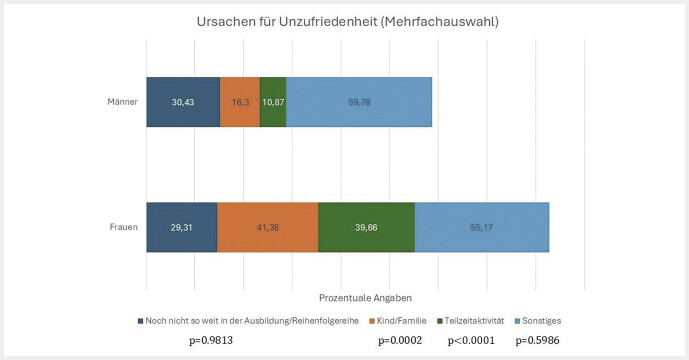
Ursachen der Unzufriedenheit unter den Befragten.

Insgesamt äußerten 70,42% der Teilnehmenden Zufriedenheit mit dem aktuellen Stand ihrer endoskopischen Tätigkeit und Ausbildung. Signifikante Unterschiede zeigten sich dabei zwischen den Geschlechtern: Während 42,38% der Frauen unzufrieden waren, traf dies nur auf 20,92% der Männer zu (p<0,0001) ( Tab. 1 und Abb. 5 ). Die höchste Unzufriedenheit wurde unter Ärztinnen und Ärzten an Universitätskliniken sowie Häusern der Maximalversorgung festgestellt (38,89% versus 36,76% versus 13,97%, p=0,6342 und p<0,0001). In diesen Versorgungsstrukturen war auch die geschlechtsspezifische Diskrepanz am ausgeprägtesten (30,56%). Demgegenüber berichteten Teilnehmende aus Praxen von der insgesamt geringsten Unzufriedenheit, sowohl in allgemeiner (13,97%) als auch in geschlechterspezifischer Hinsicht (10,00% versus 21,79%, p=0,0256) ( Tab. 2 ). Bei der Analyse der Ursachen für die Unzufriedenheit zeigten sich deutliche Unterschiede ( Abb. 6 ): Männer führten am häufigsten den noch nicht erreichten Ausbildungsstand bzw. ihre Position in der Weiterbildungsreihenfolge an (30,43%). Frauen hingegen nannten am häufigsten familiäre Verpflichtungen – insbesondere Kinderbetreuung (41,38%) – sowie Teilzeittätigkeit (39,66%) als Hauptgründe. In der Kategorie „Sonstiges“ nannten 59,78% der männlichen Befragten strukturelle Defizite wie eine unzureichende Ausbildungsorganisation, unklare oder fehlende Einarbeitung, die Priorisierung anderer Arbeitsbereiche sowie erhöhter Zeitdruck und Arbeitsverdichtung als zusätzliche Ursachen von Unzufriedenheit. Weibliche Befragte berichteten in dieser Kategorie zu 55,17% über wahrgenommene geschlechtsbezogene Diskriminierung sowie weitere geschlechtsspezifische Belastungsfaktoren. Bezüglich zukünftiger Weiterbildung äußerten die Teilnehmenden besonders großes Interesse an den Bereichen komplexer Resektionstechniken (z.B. EMR, ESD) (27,99%) sowie der therapeutischen EUS (27,65%).

### 3.5 Berufliche Zusatzaktivitäten

**Abb. 7:**
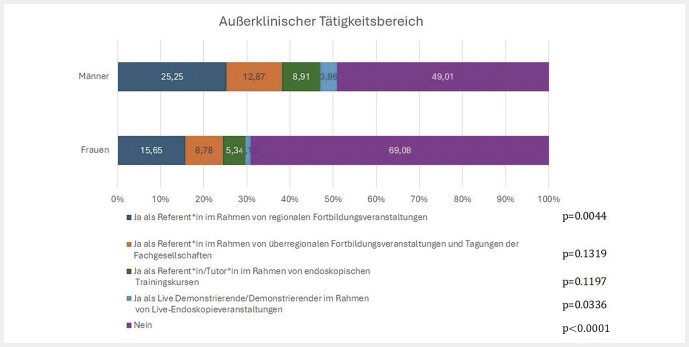
Zusätzlicher außerklinischer Tätigkeitsbereich unter den Befragten.

**Abb. 8:**
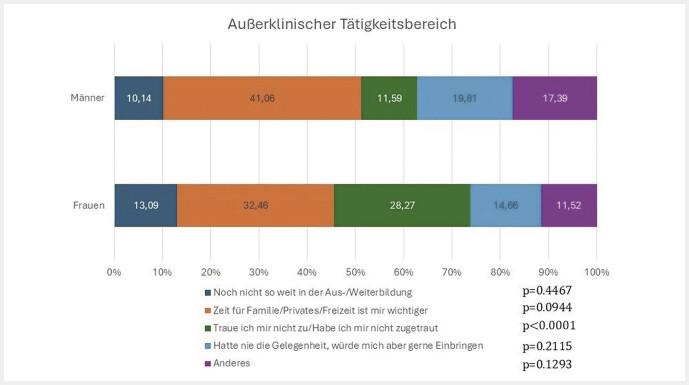
Hinderungsgründe für die Übernahme außerklinischer Tätigkeitsbereiche unter den Befragten.

**Abb. 9:**
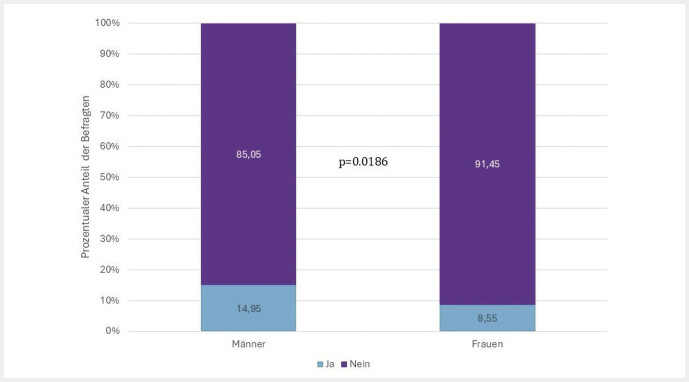
Wissenschaftliche endoskopische Tätigkeit der Befragten.

Insgesamt gaben 43,05% der Befragten an, neben ihrer klinischen Tätigkeit auch außerklinisch aktiv zu sein – etwa als Referentinnen und Referenten bei regionalen (21,52%) oder überregionalen (11,21%) Fortbildungsveranstaltungen, als Tutorinnen und Tutoren in endoskopischen Trainingskursen (7,47%) oder als Demonstrierende im Rahmen von Live-Endoskopien (2,84%). Signifikant häufiger waren Männer in diesen außerklinischen Rollen vertreten als Frauen (50,99% versus 30,92%, p<0,0001) ( Tab. 1 und Abb. 7 ). Als häufigster Grund für die Nichtteilnahme an solchen Zusatzaktivitäten wurde von beiden Geschlechtern die Priorisierung von Familie, Freizeit oder privaten Verpflichtungen angegeben (37,00%). Interessanterweise wurde dieser Grund häufiger von Männern genannt (41,08% versus 32,46%, p=0,0944), insbesondere in Universitätskliniken und Maximalversorgern (32,56% versus 13,21%, p=0,0421), wo der geschlechterspezifische Unterschied diesbezüglich am deutlichsten ausgeprägt war ( Tab. 2 ). Deutlich mehr Frauen als Männer äußerten, sich Zusatzaufgaben dieser Art fachlich oder persönlich nicht zuzutrauen (11,59% versus 28,27%, p<0,0001) ( Abb. 8 ). Der größte geschlechterspezifische Unterschied in diesem Zusammenhang zeigte sich im Bereich der Regional- und Grundversorgung (p=0,0031 versus p=0,3353 versus p=0,0132). An Universitätskliniken und Maximalversorgern wiederum berichteten Frauen im Vergleich zu Männern häufiger, grundsätzlich Interesse an außerklinischem Engagement zu haben, jedoch bislang keine entsprechende Gelegenheit erhalten zu haben (16,28% versus 32,08%, p=0,1235) – ein Unterschied, der in anderen Versorgungssektoren umgekehrt ausgeprägt war (29,11% versus 12,35%, p=0,0153 und 13,10% versus 3,57%, p=0,0754) ( Tab. 1 und Tab. 2 ). Wissenschaftlich im endoskopischen Bereich tätig waren 12,35% der Befragten und signifikant mehr Männer als Frauen (14,95% versus 8,55%, p=0,0186) ( Abb. 9 ).

### 3.6 Zukunftsperspektiven und Karrierewünsche

Bezüglich des gewünschten zukünftigen Tätigkeitsfeldes gaben 64,30% der Befragten an, im derzeitigen Versorgungssektor verbleiben zu wollen. Ein Wechsel in eine Praxis wurde von 13,19% der Teilnehmenden angestrebt, 8,30% äußerten den Wunsch, künftig an einem Haus der Maximalversorgung tätig zu sein. Ein Universitätsklinikum als zukünftigen Arbeitsort nannten insgesamt 4,59% der Befragten – dieser Wunsch wurde signifikant häufiger von Frauen als von Männern geäußert (7,52% versus 2,71%, p=0,0066) ( Tab. 1 ).

### 3.7. Subgruppenanalyse alters- und geschlechtsspezifischer Unterschiede

In einer Subgruppenanalyse der Befragung nach Altersgruppen zeigten sich deutliche Unterschiede hinsichtlich der Geschlechterverteilung, Positionen innerhalb der medizinischen Hierarchie sowie der Arbeitszufriedenheit ( Tab. 3 ).

**Table:** **Tab. 3** Vergleich der Geschlechterverteilung zwischen den Altersgruppen „jung“ (<30 Jahre/30–39 Jahre/40–49 Jahre) und „alt“ (50–59 Jahre/>60 Jahre).

Alter	<30 Jahre/30–39 Jahre/40–49 Jahre				50–59 Jahre/>60 Jahre			
	Gesamt n (%)	Männlich, n (%)	Weiblich n (%)	P-Wert	Gesamt n (%)	Männlich, n (%)	Weiblich n (%)	P-Wert
	**373 (100%)**				**311 (100%)**			
**Geschlecht (↔)**	**372 (100%)**	**189 (50,81%)**	**183 (49.19%)**	**0,7557**	**308 (100%)**	**221 (71,75%)**	**87 (28,25%)**	**0,0001**
**Position (↕)**	**372 (100%)**	**189 (100%)**	**183 (100%)**	**0,0150**	**310 (100%)**	**221 (100%)**	**86 (100%)**	**0,1145**
Chefärzt*in	18 (4,84%)	13 (6,88%)	5 (2,73%)	0,0625	60 (19,35%)	50 (22,62%)	10 (11,63%)	0,0291
Ltd Ärzt*in	34 (9,14%)	22 (11,64%)	12 (6,56%)	0,0890	49 (15,81%)	31 (14,03%)	18 (20,93%)	0,1381
Oberärzt*in	179 (48,12%)	90 (47,62%)	88 (48,09%)	1,0000	58 (18,71%)	36 (16,29%)	20 (23,26%)	0,2096
Fachärzt*in	64 (17,20%)	22 (11,64%)	42 (22,95%)	0,0059	15 (4,84%)	11 (4,98%)	4 (4,65%)	1,0000*
Assistenzärzt*in	28 (7,53%)	13 (6,88%)	15 (8,20%)	0,7754	0 (0%)	0 (0%)	0 (0%)	1,0000*
Fachärzt*in in Niederlassung	50 (13,44%)	29 (15,34%)	21 (11,48%)	0,3464	128 (41,29%)	93 (42,08%)	34 (39,53%)	0,7812
Studierende	0 (0%)	0 (0%)	0 (0%)	1,000*	0 (0%)	0 (0%)	0 (0%)	1,0000*
**Arbeitszeitmodell (↕)**	**373 (100%)**	**189 (100%)**	**183 (100%)**	**<0,0001**	**310 (100%)**	**221 (100%)**	**86 (100%)**	**0,0001**
Vollzeit	244 (65,42%)	150 (79,37%)	93 (50,82%)	<0,0001	257 (82,90%)	197 (89,14%)	60 (69,77%)	0,0001
Teilzeit	129 (34,58%)	39 (20,63%)	90 (49,18%)	<0,0001	50 (16,13%)	24 (10,86%)	26 (30,23%)	0,0001
Schichtarbeit	0 (0%)	0 (0%)	0 (0%)	1,0000*	0 (0%)	0 (0%)	0 (0%)	1,0000*
**Zufriedenheit mit aktuellen Stand der endoskopischen Tätigkeit/Ausbildung (↕)**	**373 (100%)**	**189 (100%)**	**183 (100%)**	**0,0002**	**309 (100%)**	**221 (100%)**	**87 (100%)**	**0,0203**
Ja	214 (57,37%)	127 (67,20%)	87 (47,54%)	0,0002	266 (86,08%)	197 (89,14%)	68 (78,16%)	0,0203
Nein	159 (42,63%)	62 (32,80%)	96 (52,46%)	0,0002	43 (13,92%)	24 (10,86%)	19 (21,84%)	0,0203
**Ursache für Unzufriedenheit (Mehrfachauswahl möglich) (↕)**	**161 (100%)**	**64 (100%)**	**96 (100%)**		**47 (100%)**	**28 (100%)**	**19 (100%)**	
Noch nicht so weit in der Ausbildung/Reihenfolge	59 (36,65%)	25 (39,06%)	33 (34,38%)	0,6625	4 (8,51%)	3 (10,71%)	1 (5,26%)	0,6376*
Kind/Familie	57 (35,40%)	14 (21,88%)	43 (44,79%)	0,0052	6 (12,77%)	1 (3,57%)	5 (26,32%)	0,0328*
Teilzeittätigkeit	49 (30,43%)	7 (10,94%)	42 (43,75%)	<0,0001	8 (17,02%)	4 (14,29%)	4 (21,05%)	0,6972*
Sonstiges	80 (49.69%)	31 (48,44%)	49 (51,04%)	0,8718	39 (82,98%)	24 (85,71%)	15 (78,95%)	0,6972*
**Weitere Tätigkeit neben der klinischen Tätigkeit (keine Mehrfachauswahl) (↕)**	**364 (100%)**	**185 (100%)**	**178 (100%)**	**0,0020**	**304 (100%)**	**218 (100%)**	**84 (100%)**	**0,0892**
Ja als Referent*in im Rahmen von regionalen Fortbildungsveranstaltungen	59 (16,21%)	33 (17,84%)	26 (14,61%)	0,4890	85 (27,96%)	69 (31,65%)	15 (17,86%)	0,0242
Ja als Referent*in im Rahmen von überregionalen Fortbildungsveranstaltungen und Tagungen der Fachgesellschaften	34 (9,34%)	21 (11,35%)	13 (7,30%)	0,2530	41 (13,49%)	31 (14,22%)	10 (11,90%)	0,7347
Ja als Referent*in/Tutor*in im Rahmen von endoskopischen Trainingskursen	27 (7,42%)	19 (10,27%)	8 (4,49%)	0,0579	23 (7,57%)	17 (7,80%)	6 (7,14%)	1,0000*
Ja als Live Demonstrierende/Demonstrierender im Rahmen von Live-Endoskopieveranstaltungen	7 (1,92%)	7 (3,78%)	0 (0%)	0,0149*	11 (3,62%)	8 (3,67%)	3 (3,57%)	1,0000*
Nein	237 (65,11%)	105 (56,76%)	131 (73,60%)	0,0011	144 (47,37%)	93 (42,66%)	50 (59,52%)	0,0124
**Gründe, warum nicht (↕)**	**249 (100%)**	**110 (100%)**	**138 (100%)**	**0,0020**	**151 (100%)**	**97 (100%)**	**53 (100%)**	**0,4749**
Noch nicht so weit in der Aus-/Weiterbildung	46 (18,47%)	21 (19,09%)	25 (18,12%)	0,9746	0 (0%)	0 (0%)	0 (0%)	1,0000*
Zeit für Familie/Privates/Freizeit ist mir wichtiger	75 (30,12%)	39 (35,45%)	36 (26,09%)	0,1453	73 (48,34%)	46 (47,42%)	26 (49,06%)	0,9836
Traue ich mir nicht zu/Habe ich mir nicht zugetraut	54 (21,69%)	11 (10,0%)	43 (31,16%)	0,0001	24 (15,89%)	13 (13,40%)	11 (20,75%)	0,3466
Hatte nie die Gelegenheit, würde mich aber gerne einbringen	56 (22,49%)	30 (27,27%)	25 (18,12%)	0,1163	14 (9,27%)	11 (11,34%)	3 (5,66%)	0,3802*
Anderes	18 (7,23%)	9 (8,18%)	9 (6,52%)	0,7993	40 (26,49%)	27 (27,84%)	13 (24,53%)	0,8067
**Wissenschaftliche Arbeit im Bereich der endoskopischen Forschung (↕)**	**372 (100%)**	**188 (100%)**	**183 (100%)**	**0,0042**	**307 (100%)**	**219 (100%)**	**86 (100%)**	**0,6012**
Ja	53 (14,25%)	37 (19,68%)	16 (8,74%)	0,0042	31 (10,10%)	24 (10,96%)	7 (8,14%)	0,6012
Nein	319 (85,75%)	151 (80,32%)	167 (91,26%)	0,0042	276 (89,90%)	195 (89,04%)	79 (91,86%)	0,6012
**Zukünftiger Tätigkeitsbereichswunsch (↕)**	**371 (100%)**	**188 (100%)**	**182 (100%)**	**0,0972**	**303 (100%)**	**217 (100%)**	**84 (100%)**	**0,3344**
Status idem	213 (57,41%)	112 (59,57%)	101 (55,49%)	0,4911	220 (72,61%)	155 (71,43%)	64 (76,19%)	0,4914
Universitätsklinikum	27 (7,28%)	9 (4,79%)	18 (9,89%)	0,0916	4 (1,32%)	2 (0,92%)	2 (2,38%)	0,3111*
Maximalversorger	47 (12,67%)	25 (13,30%)	21 (11,54%)	0,7224	9 (2,97%)	7 (3,23%)	2 (2,38%)	1,0000*
Grund- und Regelversorger	19 (5,12%)	10 (5,32%)	9 (4,95%)	1,0000	15 (4,95%)	12 (5,53%)	3 (3,57%)	0,5708*
Privatklinik	2 (0,54%)	2 (1,06%)	0 (0%)	0,4988*	3 (0,99)	1 (0,46%)	2 (2,38%)	0,1893*
Praxis mit stationären Belegbetten	19 (5,12%)	13 (6,91%)	6 (3,30%)	0,1800	7 (2,31%)	4 (1,84%)	3 (3,57%)	0,4033*
Praxis	44 (11,86%)	17 (9,04%)	27 (14,84%)	0,1187	45 (14,85%)	36 (16,59%)	8 (9,52%)	0,1693
(↔): prozentuale Verteilung/Berechnung innerhalb der Zeilen (↕): prozentuale Verteilung/Berechnung innerhalb der Spalten Chi-Quadrat (X ^2^ )-Test * Fischer-Exakt-Test								

**Abb. 10:**
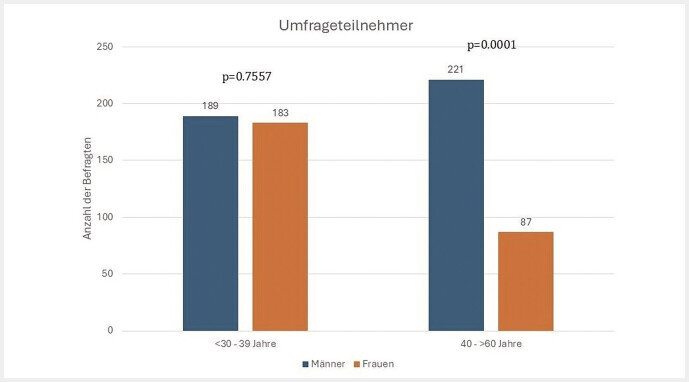
Altersspezifische Geschlechtsverteilung der Befragten.

**Abb. 11:**
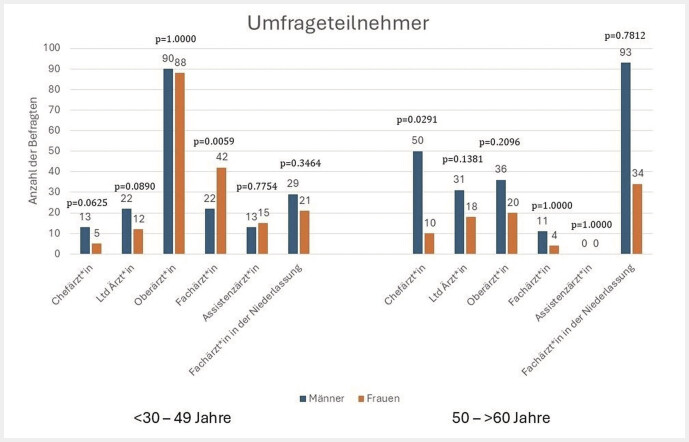
Altersspezifische berufliche Position der Befragten.

In der „jüngeren“ Altersgruppe (<30 bis 49 Jahre) war die Geschlechterverteilung nahezu ausgeglichen (189 versus 183; p=0,7955), während in der „älteren“ Gruppe (≥50 Jahre) ein Überwiegen männlicher Befragter zu verzeichnen war (71,75% versus 28,25%, p=0,0001) ( Abb. 10 ). In beiden Altersgruppen waren deutlich mehr Männer als Frauen in der Position des Chefarztes tätig (6,88% versus 2,73%, p=0,0625 und 22,62% versus 11,63%, p=0,0291). Auch in der Position der leitenden Ärztin bzw. des leitenden Arztes zeigte sich in der jüngeren Gruppe ein männlicher Überhang (11,64% verus 6,56%, p=0,0890), während in der älteren Gruppe ein höherer Frauenanteil verzeichnet wurde (14,03% versus 20,93%, p=0,1381). Im Hinblick auf die Position der Oberärztinnen und Oberärzte war in der älteren Gruppe ein höherer Frauenanteil festzustellen (16,29% versus 23,36%, p=0,2096), während in der jüngeren Gruppe eine ausgeglichene Geschlechterverteilung vorlag (47,62% versus 48,09%, p=1,0000). Den Facharztstatus hatten in der jüngeren Altersgruppe signifikant mehr Frauen als Männer erreicht (11,64% versus 22,95%, p=0,0059), in der älteren Gruppe zeigte sich hier kein signifikanter Unterschied (4,98% versus 4,65%, p=1,0000). Auch unter den niedergelassenen Fachärztinnen und Fachärzten war die Verteilung geschlechtsunabhängig und altersgruppenübergreifend vergleichbar (p=0,3464 und p=0,7812) ( Abb. 11 ).

**Abb. 12:**
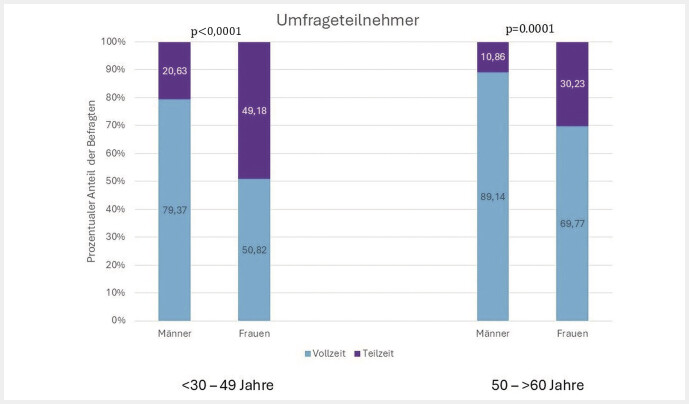
Altersspezifisches Arbeitszeitmodell der Befragten.

Signifikant häufiger waren Teilzeittätigkeiten in der jüngeren Altersgruppe vertreten (34,58% versus 16,13%, p<0,0001) ( Abb. 12 ). Besonders ausgeprägt war dies bei den Frauen: Während knapp 50% der jüngeren weiblichen Befragten in Teilzeit arbeiteten, traf dies nur auf 30,23% ihrer älteren Kolleginnen zu. Der geschlechterspezifische Unterschied in der Teilzeitarbeit betrug in der jüngeren Gruppe etwa 30%, in der älteren Gruppe rund 20%.

**Abb. 13:**
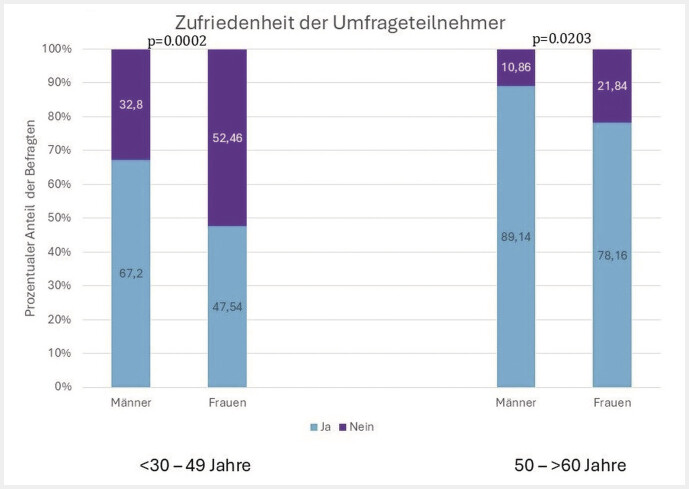
Altersspezifische Zufriedenheit der Befragten mit der aktuellen endoskopischen Tätigkeit und Ausbildung.

Deutlich häufiger äußerten Befragte der jüngeren Altersgruppe Unzufriedenheit mit dem Stand ihrer Ausbildung und der endoskopischen Tätigkeit (42,62% versus 13,92%, p<0,0001) ( Abb. 13 ). Besonders betroffen waren jüngere Ärztinnen: Über 50% von ihnen gaben an, unzufrieden zu sein, verglichen mit 32,80% der männlichen Kollegen in derselben Altersgruppe und 21,84% der älteren Kolleginnen. Als Ursachen wurden in der jüngeren Gruppe am häufigsten „noch so nicht weit in der Ausbildung/Reihenfolge“ (36,65%), „Vereinbarkeit von Beruf und Familie“ (35,40%) sowie „Teilzeittätigkeit“ (30,43%) genannt. Über alle Altersgruppen hinweg nannten signifikant mehr Frauen als Männer familiäre Verpflichtungen (21,88% versus 44,79%, p=0,0052 und 3,57% versus 26,32%, p=0,0328) und Teilzeitarbeit (10,94% versus 43,75%, p<0,0001 und 14,29% versus 21,05%, p=0,6972) als Gründe für ihre Unzufriedenheit.

Jüngere Befragte waren häufiger ausschließlich klinisch tätig und weniger in außerklinischen Aktivitäten engagiert (65,11% versus 47,37%, p<0,0001). Die Beteiligung an Tutorentätigkeiten in endoskopischen Trainingskursen oder als Demonstratoren bei Live-Endoskopie-Workshops war in beiden Altersgruppen gering. Während sich in der älteren Gruppe eine geschlechtsneutrale Verteilung zeigte (p<0,0001 und p<0,0001), waren in der jüngeren Gruppe mehr Männer in diesen Funktionen vertreten (10,27% versus 4,49%, p=0,0579 und 3,78% versus 0%, p=0,0149).

Befragte ohne zusätzliche Tätigkeiten gaben in beiden Altersgruppen als häufigsten Grund die Priorisierung von Familie, Freizeit und privaten Interessen an (30,12% und 47,42%). In der jüngeren Altersgruppe gaben signifikant mehr Frauen an, sich zusätzliche Aufgaben – insbesondere in wissenschaftlichen oder lehrenden Funktionen – nicht zuzutrauen (10,0% versus 31,16%, p=0,0001).

Die Beteiligung an wissenschaftlicher endoskopischer Tätigkeit war in der jüngeren Altersgruppe insgesamt höher (14,25% versus 10,96%, p=0,1022), wobei Männer in der jüngeren Altersgruppe signifikant häufiger wissenschaftlich endoskopisch tätig waren als Frauen (19,68% versus 8,74%, p=0,0042).

Bezüglich der beruflichen Zukunft zeigten sich altersabhängige Unterschiede: Während 72,61% der älteren Befragten angab, im aktuellen klinischen Tätigkeitsbereich verbleiben zu wollen (Status idem), äußerten nur 57,41% der jüngeren Gruppe diesen Wunsch (p<0,0001). Jüngere Befragte nannten neben dem Status idem als bevorzugte zukünftige Arbeitsorte insbesondere Maximalversorger (12,67%) und Praxen (11,86%), während ältere Befragte vorrangig eine Tätigkeit in der Praxis (14,85%) anstrebten.

### 3.8. Multivariate binäre logistische Regressionsmodelle

**Abb. 14:**
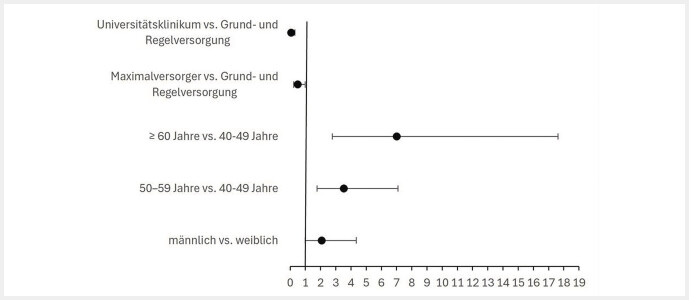
Forest Plot der adjustierten Odds Ratios (OR) mit 95%-Konfidenzintervallen aus multivariaten logistischen Regressionsmodell zu Führungsposition.

**Abb. 15:**
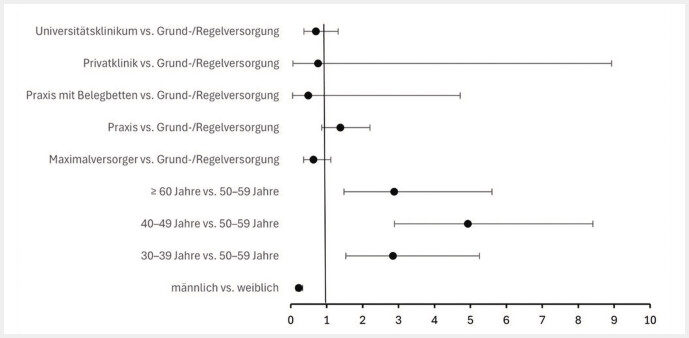
Forest Plot der adjustierten Odds Ratios (OR) mit 95%-Konfidenzintervallen aus multivariaten logistischen Regressionsmodell zu Teilzeittätigkeit.

**Abb. 16:**
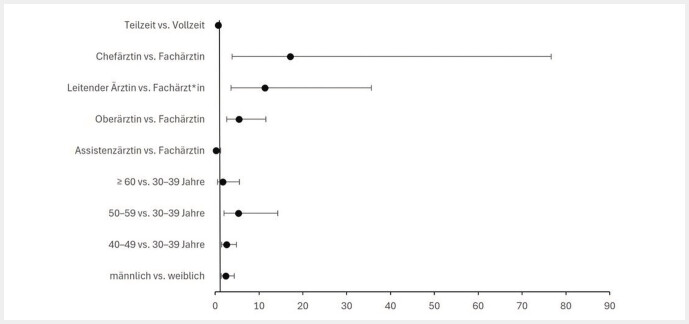
Forest Plot der adjustierten Odds Ratios (OR) mit 95%-Konfidenzintervallen aus multivariaten logistischen Regressionsmodell zu komplexer endoskopischer Tätigkeit.

Über alle drei multivariaten binären logistischen Regressionsmodelle hinweg zeigten sich konsistente Zusammenhänge zwischen Geschlecht, Alter und beruflicher Stellung mit den untersuchten beruflichen Zielvariablen ( Tab. 4 , Tab. 5 , Tab. 6 und Abb. 14 , Abb. 15 , Abb. 16 ).

**Table:** **Tab. 4** Multivariate binomiale logistische Regressionsanalyse mit Odds Ratios (OR) und 95%-Konfidenzintervallen zur Assoziation von Geschlecht, Alter, Arbeitsort und Arbeitszeit mit einer Führungsposition (Chefärzt*innenstatus).

**Referenzkategorien:** weiblich, 40–49 Jahre, Grund- und Regelversorgung, Vollzeit

**Stichprobe:** N = 311.

Prädiktor	Odds Ratio (OR)	95 %-KI	p-Wert
**Geschlecht**			
Männlich vs. weiblich	2,08	0,99–4,35	0,05
**Alter**			
50–59 Jahre vs. 40–49 Jahre	3,53	1,76–7,09	<0,001
<30 Jahre vs. 40–49 Jahre	<0,01	0,00–∞	0,997
≥60 Jahre vs. 40–49 Jahre	7	2,78–17,62	<0,001
**Arbeitsort**			
Maximalversorger vs. Grund- und Regelversorgung	0,49	0,24–0,99	0,047
Privatklinik vs. vs. Grund- und Regelversorgung	<0,01	0,00–∞	0,996
Universitätsklinikum vs. vs. Grund- und Regelversorgung	0,07	0,01–0,31	<0,001
**Arbeitszeit**			
Teilzeit vs. Vollzeit	<0,01	0,00–∞	0,987
**Modellgüte:** AIC = 260; McFadden R² = 0,310			

**Table:** **Tab. 5** Multivariate binomiale logistische Regressionsanalyse mit Odds Ratios (OR) und 95%-Konfidenzintervallen zur Assoziation von Geschlecht, Alter und Arbeitsort mit Arbeitszeit.

**Referenzkategorien:** weiblich, 50–59 Jahre, Grund- und Regelversorgung

**Stichprobe:** N = 676.

Prädiktor	Odds Ratio (OR)	95 %-KI	p-Wert
**Geschlecht**			
Männlich vs. weiblich	0,22	0,15–0,33	<0,001
**Alter**			
30–39 Jahre vs. 50–59 Jahre	2,84	1,53–5,25	<0,001
40–49 Jahre vs. 50–59 Jahre	4,93	2,89–8,41	<0,001
≥60 Jahre vs. 50–59 Jahre	2,88	1,48–5,60	0,002
<30 Jahre vs. 50–59 Jahre	n. s.	–	0,984
**Arbeitsort**			
Maximalversorger vs. Grund- und Regelversorgung	0,63	0,36–1,12	0,114
Praxis vs. Grund-/Regelversorgung	1,38	0,86–2,21	0,178
Praxis mit Belegbetten vs. Grund-/Regelversorgung	0,49	0,05–4,72	0,535
Privatklinik vs. Grund-/Regelversorgung	0,76	0,06–8,93	0,828
Universitätsklinikum vs. vs. Grund- und Regelversorgung	0,7	0,37–1,32	0,269
**Modellgüte:** AIC = 691; McFadden R²= 0.147			

**Table:** **Tab. 6** Multivariate binomiale logistische Regressionsanalyse mit Odds Ratios (OR) und 95%-Konfidenzintervallen zur Assoziation von Geschlecht, Alter, Position und Arbeitszeit mit der Ausübung komplexer endoskopischer Tätigkeiten.

**Referenzkategorien:** weiblich, 30–39 Jahre, Fachärzt*in, Vollzeit

**Stichprobe:** N = 452.

Prädiktor	Odds Ratio (OR)	95 %-KI	p-Wert
**Geschlecht**			
Männlich vs. weiblich	2,48	1,42–4,34	0,001
**Alter**			
40–49 vs. 30–39 Jahre	2,66	1,45–4,88	0,002
50–59 vs. 30–39 Jahre	5,35	2,00–14,27	<0,001
≥60 vs. 30–39 Jahre	1,75	0,55–5,53	0,344
**Position**			
Assistenzärzt *in vs. Fachärzt* in	0,24	0,05–1,24	0,089
Oberärzt *in vs. Fachärzt* in	5,49	2,61–11,55	<0,001
Leitende *r Ärzt* in vs. Fachärzt*in	11,35	3,62–35,60	<0,001
Chefärzt *in vs. Fachärzt* in	17,15	3,84–76,62	<0,001
Arbeitszeit			
Teilzeit vs. Vollzeit	0,71	0,39–1,30	0,265
**Modellgüte:** McFadden R² = 0,32			

Geschlecht war in allen Modellen ein relevanter Prädiktor: Männliche Ärzte wiesen signifikant höhere Odds auf, eine Führungsposition (Chefarzt*in) innezuhaben (OR = 2,08; 95-%-KI 0,99–4,35; p=0,05) sowie komplexe endoskopische Tätigkeiten durchzuführen (OR = 2,48; 95 % KI 1,42–4,34; p=0,001). Demgegenüber war männliches Geschlecht mit einer signifikant geringeren Wahrscheinlichkeit für Teilzeittätigkeit assoziiert (OR = 0,22; 95%-KI: 0,15–0,33; p<0,001).

Alter zeigte je nach abhängiger Variable unterschiedliche Muster. Die Wahrscheinlichkeit einer Führungsposition (50–59 Jahre (OR = 3,53; 95%-KI: 1,76–7,09; p<0,001); ≥60 Jahren (OR = 7; 95%-KI:2,78–17,62; p<0,001)) sowie der Durchführung komplexer endoskopischer Tätigkeiten nahm mit steigendem Alter zu und erreichte ihr Maximum in den Altersgruppen zwischen 50 und 59 Jahren (40–49 Jahren (OR = 2,66; 95%-KI:1,45–4,88; p=0,002); 50–59 Jahren (OR = 5,35; 95%-KI: 2,0–14,27; p<0,001)). Im Gegensatz dazu zeigte sich für Teilzeittätigkeit ein altersabhängiges Umkehrmuster mit der höchsten Prävalenz in der Altersgruppe der 30–39-Jährigen (OR = 2,84; 95%-KI: 1,53–5,25; p<0,001).

Berufliche Position war der stärkste Prädiktor für die Durchführung komplexer endoskopischer Tätigkeiten. Oberärzt*innen (OR = 5,49; 95%-KI: 2,61–11,55), leitende Ärzt*innen (OR = 11,35; 95%-KI: 3,62–35,60) und Chefärzt*innen (OR = 17,15; 95%-KI: 3,84–76,62) hatten gegenüber Fachärztinnen signifikant höhere Odds (alle p<0,001), komplexe endoskopische Prozeduren durchzuführen.

Arbeitszeitmodelle zeigten differenzierte Effekte: Während Teilzeittätigkeit stark mit Geschlecht und Alter assoziiert war, zeigte sich nach Adjustierung kein unabhängiger Effekt der Teilzeitarbeit auf die Durchführung komplexer endoskopischer Tätigkeiten (p=0,265).

Der Arbeitsort war insbesondere für die Führungsposition relevant. Ärzt*innen an Universitätskliniken (OR = 0,07; 95%-KI: 0,01–0,31 p<0,001) und Maximalversorger (OR = 0,49; 95%-KI: 0,24–0,99 p<0,047) wiesen im Vergleich zur Grund- und Regelversorgung eine signifikant geringere Odds auf, eine Chefärzt*innenposition zu bekleiden. Der Arbeitsort war insgesamt nicht signifikant mit Teilzeitarbeit assoziiert (alle p>0,05).

Die erklärten Varianzanteile der Modelle variierten zwischen niedrig (Teilzeittätigkeit) und moderat bis hoch (Führungsposition und komplexe Endoskopie), was auf unterschiedliche strukturelle Determinanten der jeweiligen Zielvariablen hinweist.

## 4. Diskussion

In den letzten Jahren ist das Interesse an der Rolle der Frau im medizinischen Beruf sowie ihrer beruflichen Entwicklung deutlich gewachsen. Die steigende Zahl an weiblichen Medizinstudentinnen nährt die Hoffnung auf eine zunehmende Gleichstellung der Geschlechter in allen Bereichen der medizinischen Laufbahn. Trotz dieser positiven Entwicklung ist der Anteil von Frauen in leitenden und akademischen Positionen, insbesondere im Fachbereich der Gastroenterologie, nach wie vor deutlich geringer als der ihrer männlichen Kollegen 6 7 . Diese anhaltenden Geschlechterunterschiede in Ausbildung, Karriereentwicklung und der Vereinbarkeit von Beruf und Privatleben wurden in jüngerer Zeit in mehreren Publikationen 8 9 thematisiert, unter anderem in einem Positionspapier der European Society of Gastrointestinal Endoscopy (ESGE) 10 . Die vorliegende Umfrage zielt darauf ab, die aktuelle Situation in Deutschland im Hinblick auf geschlechterspezifische Unterschiede innerhalb der Gastroenterologie abzubilden und mögliche Ursachen für bestehende Disparitäten zu identifizieren.

Die Ergebnisse zeigen eine weiterhin bestehende strukturelle Ungleichheit: So befinden sich lediglich 5,58% der befragten Frauen in einer Chefarztposition, im Vergleich zu 15,33% der Männer (p=0,0002). Von den insgesamt 78 Befragten in Chefarztpositionen entspricht dies einem Verhältnis von 81% Männern zu 19% Frauen (p<0,0001). Ein vergleichbares Muster zeigt sich auf der Ebene der leitenden Ärztinnen und Ärzte, auf der ein Anteil von 64% Männern gegenüber 36% Frauen festgestellt wurde. Demgegenüber akkumuliert sich der Anteil der weiblichen Fachkräfte überwiegend auf den Ebenen der Oberärztinnen und Fachärztinnen. Diese Ergebnisse dieser Umfrage unterstreichen eine nach wie vor bestehende, signifikante Unterrepräsentation von Frauen in Führungspositionen innerhalb der deutschen Gastroenterologie. Auch nach Berücksichtigung von Alter, Arbeitsort und Arbeitszeitmodell bleibt das Geschlecht ein unabhängiger Prädiktor für das Erreichen einer Chefärzt*innenposition.

Diese Ergebnisse stehen im Einklang mit internationalen Daten. Eine von Ciacci et al. durchgeführte Studie mit 564 Befragten ergab, dass unter 113 Gastroenterolog*innen in Führungspositionen lediglich 26 Frauen vertreten waren 11 . Ebenso zeigte eine Untersuchung von Jawaid et al. (2020), dass Frauen in allen Stufen der gastroenterologischen Karriere unterrepräsentiert sind: Zwischen 2018 und 2020 lag der Anteil von Frauen, die eine leitende Position in GI-Abteilungen akademischer Einrichtungen innehatten, zwischen 0% und 13%. Der Anteil weiblicher Programmleiterinnen betrug im gleichen Zeitraum lediglich 29% bis 36% 12 . Auch in den USA spiegeln sich diese Ungleichheiten wider: Eine 2021 durchgeführte Studie mit 3.655 Fakultätsmitgliedern und Auszubildenden an 163 akademischen gastroenterologischen Programmen zeigte, dass nur 19,4% der 289 Führungspersonen weiblich waren 13 .

Es ließe sich argumentieren, dass die geschlechterspezifische Ungleichverteilung in medizinischen Führungspositionen in den kommenden Jahren allein durch den demografischen Wandel ausgeglichen werde. Tatsächlich ist in den vergangenen Jahren ein kontinuierlicher Anstieg des Anteils weiblicher Medizinstudierender zu beobachten – mittlerweile übertrifft dieser den männlichen Anteil deutlich 1 . Die Ergebnisse unserer aktuellen Erhebung zeigen jedoch, dass eine rein zahlenmäßige Zunahme von Frauen in der Medizin nicht ausreicht, um das sogenannte *Leaky-Pipeline* -Phänomen zu durchbrechen. In der altersstratifizierten Analyse unserer Umfrage war die Geschlechterverteilung in der jüngeren Kohorte nahezu ausgeglichen, wohingegen in der älteren Kohorte ein Überwiegen männlicher Befragter festzustellen war. Dieses Muster spiegelt die demografische Entwicklung der vergangenen Jahrzehnte wider. Trotz dieses Fortschritts zeigen sich jedoch in beiden Altersgruppen weiterhin große geschlechterspezifische Unterschiede in der Verteilung von Führungspositionen. Insbesondere in der Chefarztposition ist der Männeranteil in beiden Altersgruppen wesentlich höher. Auch in der jüngeren Alterskohorte zeigt sich ein deutlicher Männerüberschuss in leitenden ärztlichen Funktionen. Bemerkenswert ist dabei, dass in der jüngeren Altersgruppe 2,5-mal mehr Männer in der Chefarztposition vertreten sind, während dieses Verhältnis in der älteren Gruppe bei 1,9 liegt. Diese Diskrepanz deutet darauf hin, dass sich bestehende strukturelle Barrieren offenbar auch in der nachrückenden Generation fortsetzen. Diese Ergebnisse verdeutlichen, dass der bloße Anstieg des Frauenanteils in der Medizin – sei es im Studium oder im Berufseinstieg – nicht ausreicht, um die bestehende Geschlechterungleichheit auf Führungsebene zu beheben. Demografische Veränderungen allein scheinen nicht auszureichen, um die strukturelle Ungleichheit zu beseitigen; vielmehr sind gezielte institutionelle Maßnahmen erforderlich.

Quotenbasierte Maßnahmen gelten vielfach als ein Instrument zur Förderung von Gleichstellung in Führungs- und Ausbildungspositionen. Tatsächlich zeigen empirische Daten, dass verbindliche Quotenmodelle sowohl zu einer gesteigerten Wahrnehmung von Ungleichheiten („Awareness“) als auch zu einer Zunahme qualifizierter Bewerbungen führen können. Eine norwegische Studie belegte beispielsweise, dass die Einführung einer verbindlichen 40%-Quote zu einem Anstieg der Bewerbungen qualifizierter Frauen um rund 30% führte 14 . Ähnlich zeigen Erfahrungen aus dem österreichischen Gastroenterologie und Hepatologie (GEH)-Facharztsystem, dass sich der Frauenanteil durch die Einführung transparenter Auswahlkriterien zwischen 2015 und 2020 signifikant von 40% auf 55% steigern ließ 6 .

Trotz dieser positiven Entwicklungen sind quotenbasierte Maßnahmen nicht frei von Risiken und Nachteilen. Besonders hervorzuheben ist hierbei das Phänomen des *Tokenismus* , bei dem Mitglieder unterrepräsentierter Gruppen in symbolischer Weise in dominante Strukturen integriert werden, ohne dass sich bestehende Machtverhältnisse substanziell verändern. Individuen mit sogenanntem „Token-Status“ geraten dadurch häufig verstärkt ins berufliche und soziale Rampenlicht. Ihre Leistungen werden überproportional beobachtet, bewertet und kritisiert, was zu einem erhöhten Leistungsdruck und einer messbar höheren Arbeitsbelastung führen kann – Studien berichten hier von einer Zunahme des Workloads um bis zu 15% 14 . Ein konkretes Beispiel bietet die Ausbildung in der „Advanced Endoscopy“: Nach einer plötzlichen Erhöhung des Frauenanteils von 30% auf 42% wurde ebenso ein Anstieg der Abbruchquoten festgestellt 15 . Auch psychische Belastungen können infolge solcher Dynamiken zunehmen. Eine schwedische Untersuchung zeigte, dass in den ersten drei Jahren nach Einführung einer Frauenquote die Burnout-Rate um 22% anstieg 14 . Darüber hinaus kann der durch Quoten vermittelte Token-Status bei den Betroffenen zu Zweifeln an der eigenen Legitimation führen. In einer Umfrage berichteten 27% der befragten Chefärztinnen, dass sie ihre Position durch Dritte infrage gestellt sahen – allein aufgrund ihres Geschlechts und der unterstellten Förderung durch eine Quote 16 . Dieser Rechtfertigungsdruck kann wiederum zur *Assimilation* führen, bei der sich Angehörige unterrepräsentierter Gruppen den Normen der dominanten Gruppe anpassen – oft zulasten der Identifikation mit der eigenen Gruppe. Dies kann die soziale Integration und den Zusammenhalt innerhalb der Minderheitengruppe nachhaltig beeinträchtigen. Nicht zuletzt sind auch unbeabsichtigte Rückwirkungen auf die Mehrheitsgruppe zu berücksichtigen: Bis zu 40% der männlichen Befragten gaben an, sich durch Quotenregelungen benachteiligt zu fühlen 17 – was die Akzeptanz und langfristige Wirksamkeit solcher Maßnahmen zusätzlich gefährden kann. Diese Ergebnisse machen deutlich, dass Quotenregelungen zwar wichtige Impulse setzen können, jedoch differenziert betrachtet und durch begleitende Maßnahmen flankiert werden müssen, um sowohl strukturelle Gleichstellung als auch individuelle Chancengleichheit nachhaltig zu fördern.

Die Thematik der beruflichen Gleichstellung der Geschlechter erweist sich als deutlich komplexer und vielschichtiger, und sollte durch rein zahlenbasierte Maßnahmen wie die Einführung von Frauenquoten nicht von einer differenzierten Auseinandersetzung mit den tatsächlichen Gründen und Barrieren, die Frauen auf dem Weg in Führungspositionen begegnen, ablenken. Vor diesem Hintergrund stellt sich die zentrale Frage: Welche strukturellen, institutionellen und gesellschaftlichen Faktoren behindern nach wie vor eine gleichberechtigte Teilhabe von Frauen an Führungspositionen, Aufstiegsmöglichkeiten und Karrierechancen im Fachbereich der Gastroenterologie?

In unserer Umfrage wurden die Teilnehmenden zu Umfang und Art ihres endoskopischen Tätigkeitsbereiches befragt. Es zeigte sich, dass Männer im Durchschnitt deutlich länger endoskopisch tätig waren als Frauen. Während Basisuntersuchungen – wie die diagnostische ÖGD, Koloskopie und EUS – von beiden Geschlechtern in vergleichbarer Häufigkeit durchgeführt wurden, bestand bei komplexen endoskopischen Interventionen ein erheblicher geschlechtsspezifischer Unterschied. Maßnahmen wie EMR von Läsionen >2 cm, ESD, Zenkerdivertikulotomien, therapeutische EUS sowie einfache und komplexe ERCP wurden von Männern bis zu 20% häufiger durchgeführt. Eine US-amerikanische Erhebung ergab, dass 31,6% der *advanced endoscopy* -Programme noch nie eine Frau aufgenommen hatten 15 . Im Jahr 2019 wurden lediglich 12,8% der *advanced endoscopy fellowship* -Plätze in den USA von Frauen besetzt; wobei 47,4% der Programme über keine weiblichen Mitglieder verfügten 15 . Dies legt nahe, dass geschlechtsspezifische Stereotype nicht nur die Wahl des medizinischen Fachgebiets, sondern auch den Tätigkeitsbereich innerhalb eines Fachgebiets beeinflussen.

Die Gastroenterologie – ähnlich wie chirurgische Disziplinen – ist häufig mit Attributen belegt, die traditionell als „männlich“ gelten, wie handwerkliches Geschick, körperliche Kraft, Ausdauer, Objektivität, emotionale Distanz, Autorität und Wettbewerbsorientierung 18 19 20 . Diese Zuschreibungen können dazu führen, dass Frauen diese Fachrichtung seltener wählen; so meiden beispielsweise 78% der Medizinstudentinnen chirurgische Fächer 21 . Komplexe endoskopische Verfahren werden oftmals mit diesen männlich konnotierten Eigenschaften assoziiert, wodurch Frauen deren Durchführung weniger zugetraut wird. Dies kann zu einer geschlechtsspezifischen Arbeitsteilung führen und die bestehende horizontale wie auch vertikale Segregation verstärken.

Zusätzlich spielen möglicherweise Bedenken hinsichtlich Schwangerschaft und potenzieller Strahlenbelastung während endoskopischer Eingriffe eine Rolle für die Unterrepräsentation von Frauen in Führungspositionen sowie in der *advanced endoscopy* 22 . Strahlenexposition wird auch in anderen Fachbereichen – wie der Interventionskardiologie und der Interventionsradiologie – als Hindernis wahrgenommen. In einer Studie aus dem Jahr 2016 wurde sie als der am häufigsten genannte Grund angegeben, weshalb Medizinstudentinnen von einer Weiterbildung in der Interventionsradiologie Abstand nahmen 23 .

Darüber hinaus könnten ergonomische Faktoren die Ausübung komplexer endoskopischer Verfahren durch Frauen erschweren. Standardisierte Endoskope nach dem Prinzip „one size fits all“ berücksichtigen nicht geschlechtsspezifische Unterschiede in Handgröße und Kraft. Es konnte gezeigt werden, dass die Handgröße die Lernkurve in der Endoskopie beeinflusst. Korrespondierend berichten Gastroenterologinnen häufiger als ihre männlichen Kollegen über muskuloskelettale Beschwerden 24 .

Auch ein Mangel an weiblichen Vorbildern und Mentorinnen könnte sowohl die vertikale als auch die horizontale Segregation in der Gastroenterologie verstärken 15 25 . Umgekehrt korreliert ein höherer Frauenanteil in Führungspositionen mit einem Anstieg des Anteils weiblicher Programmleitungen sowie einer Zunahme weiblicher Nachwuchskräfte in der Ausbildung 26 . In unserer Umfrage gaben signifikant mehr männliche als weibliche Teilnehmende an, außerklinisch als Referenten bei regionalen oder überregionalen Fortbildungsveranstaltungen und Kongressen tätig zu sein. Ebenso waren häufiger Männer als Tutoren in endoskopischen Trainingskursen oder als Demonstratoren bei Live-Endoskopien vertreten. Eine mögliche zentrale Ursache für diese Unterschiede könnte die weiterhin unzureichende Präsenz weiblicher Vorbilder und Mentorinnen in der außerklinischen gastroenterologischen Fachlandschaft darstellen.

In der Umfrage wurden die Teilnehmenden auch zur Zufriedenheit mit dem Stand ihrer Ausbildung und ihrer endoskopischen Tätigkeit befragt. Frauen äußerten signifikant häufiger Unzufriedenheit als Männer. Besonders ausgeprägt war die Unzufriedenheit bei Ärztinnen und Ärzten an Universitätskliniken sowie an Häusern der Maximalversorgung und in der jüngeren Altersgruppe. Bemerkenswert ist, dass 52,46% der jüngeren Ärztinnen unzufrieden waren. Als Hauptgründe für die Unzufriedenheit nannten 81% der befragten Frauen familiäre Verpflichtungen, Kinderbetreuung sowie Teilzeittätigkeit – im Vergleich zu nur 27% der Männer. Trotz des steigenden Anteils weiblicher Medizinstudierender und Ärztinnen hat sich an der tradierten Rollenverteilung wenig geändert: Die Hauptverantwortung für Kinder und Familie sowie die damit oft verbundene Teilzeitarbeit liegt weiterhin überwiegend bei Frauen. Zwar nimmt Teilzeitarbeit bei beiden Geschlechtern in der jüngeren Altersgruppe zu, der geschlechtsspezifische Unterschied bleibt jedoch bestehen. Die Vereinbarkeit von Beruf und Privatleben wird häufig als zentrales Hindernis für eine ausgewogene Geschlechterverteilung in Spitzenpositionen genannt 15 25 27 . Frauen müssen deutlich häufiger als Männer zwischen Karriere, Familie und Kindern abwägen und ordnen ihre beruflichen Ziele oft den familiären Bedürfnissen unter 12 . Dies führt zu eingeschränkten Aufstiegsmöglichkeiten und verminderten Karriereperspektiven 28 . Studien belegen diese Diskrepanz: In einer landesweiten Befragung zeigten Jolly et al., dass Frauen im Durchschnitt 8,5 Stunden pro Woche mehr für häusliche Tätigkeiten aufwenden als ihre (Ehe-)Partner 29 . Weitere Untersuchungen berichten, dass von Frauen nach wie vor erwartet wird, den Großteil der Kindererziehung und Haushaltsorganisation zu übernehmen 18 . Diese gesellschaftliche Akzeptanz der ungleichen Aufgabenverteilung sowie die ungleiche Bewertungsskala dieser Aufgabenverteilung führt dazu, dass Frauen Karrieremöglichkeiten verpassen, während ein Rollenwechsel gesellschaftlich noch immer nicht vollständig anerkannt ist.

Dabei sind diese Rollenbilder nicht biologisch determiniert, sondern sozial konstruiert und werden bereits in der frühen Kindheit vermittelt. Kinder verinnerlichen geschlechtsspezifische Erwartungen durch familiäre Vorbilder, geschlechtsbezogene Spielzeuge, schulische Erziehung sowie mediale Darstellung 30 . So werden Eigenschaften wie Ordnungssinn und Fürsorglichkeit bei Mädchen als selbstverständlich vorausgesetzt und unentgeltliche Übernahme solcher Tätigkeiten als „natürlich“ dargestellt. Diese verinnerlichten und gesellschaftlich anerzogenen Rollenbilder wirken einer tatsächlichen beruflichen Gleichstellung entgegen und tragen zu einer geschlechtsspezifischen Segregation im medizinischen Berufsalltag bei 31 .

Nicht nur tradierte Rollenbilder, sondern auch tief verankerte geschlechtsspezifische Stereotype stellen wesentliche Hürden für die Gleichberechtigung auf allen Ebenen der beruflichen Laufbahn dar. Männern werden in diesem Kontext häufig Eigenschaften wie Mut, Durchsetzungsvermögen, Lautstärke, körperliche Stärke, Selbstbewusstsein und Wettbewerbsorientierung zugeschrieben. Weibliche Stereotype hingegen sind oftmals mit Attributen wie Feinfühligkeit, Fürsorglichkeit, Sanftheit, Freundlichkeit, Einfühlungsvermögen, Zurückhaltung, Vorsicht und Gemeinschaftsorientierung verknüpft. Diese Zuschreibungen führen dazu, dass Männer und Frauen in vergleichbaren beruflichen Positionen und Führungsrollen unterschiedlich bewertet werden 32 . In einer Studie von Kolehmainen et al. zur Wahrnehmung von Durchsetzungsvermögen gaben sowohl Männer als auch Frauen an, dass beide Geschlechter gleichermaßen gute Führungspersönlichkeiten sein können. Auffällig war jedoch, dass sich deutlich mehr Frauen unwohl damit fühlten, sich offen und bestimmt zu äußern, da sie befürchteten, als „herrisch“ wahrgenommen zu werden 33 . Fassiotto et al. zeigten zudem, dass weibliche Lehrkräfte in der akademischen Medizin häufig einen Konflikt zwischen den stereotypen Vorstellungen von „weiblich“ und den Stereotypen einer „Führungspersönlichkeit“ erleben 34 . Auch Stellenausschreibungen führender medizinischer Fakultäten enthalten überproportional häufig Formulierungen mit stereotyp „männlichen“ Attributen im Vergleich zu „weiblichen“ oder neutralen Begriffen 35 . Der Einfluss dieser Stereotype beschränkt sich nicht allein auf die externe Bewertung, sondern prägt auch die internalisierte, gesellschaftlich erlernte Selbstwahrnehmung. Entsprechend gaben überproportional viele Frauen an, sich außerklinische Tätigkeiten – etwa als Referentinnen bei regionalen oder überregionalen Fortbildungsveranstaltungen und Kongressen, als Tutorinnen in endoskopischen Trainingskursen oder als Demonstratorinnen bei Live-Endoskopien – nicht zuzutrauen.

Diese geschlechtsspezifischen Stereotype sind weder genetisch bedingt noch angeboren, sondern werden früh in der Sozialisation vermittelt und über gesellschaftliche Strukturen verstärkt.

Frauen nehmen in der Gastroenterologie jedoch eine zentrale Rolle ein. Mehrere Studien belegen, dass insbesondere Patientinnen für ambulante Konsultationen und Koloskopien häufig gezielt weibliche Gastroenterologinnen aufsuchen 36 . Neben dem grundsätzlichen Ziel der Gleichberechtigung zeigen Untersuchungen, dass Quotenregelungen und eine ausgewogene Zusammensetzung geschlechtergemischter Teams die Qualität der Patientenversorgung verbessern können 15 . So wurde beispielsweise in einer Analyse chirurgischer Eingriffe festgestellt, dass Patientinnen und Patienten, die von Chirurginnen operiert wurden, sowohl 90 Tage als auch ein Jahr postoperativ signifikant weniger Komplikationen aufwiesen als jene, die von männlichen Operateuren behandelt wurden 37 . Darüber hinaus bringen Frauen in Führungspositionen nachweislich zusätzliche Vorteile für Organisationen mit sich. Sie fokussieren sich häufiger auf kollaborative, langfristige Zielsetzungen und tragen dadurch zu einer Verbesserung sowohl der organisatorischen als auch der finanziellen Leistungsfähigkeit bei 17 38 .

Vor diesem Hintergrund stellt sich die Frage, an welchen strukturellen und gesellschaftlichen Stellschrauben anzusetzen ist, um eine tatsächliche geschlechtliche Gleichberechtigung auf allen Ebenen der beruflichen Karriere in der Medizin zu erreichen.

Allgemeine Maßnahmen wie flexible Arbeitszeiten sowie die Bereitstellung ausreichender Kinderbetreuungsplätze sowohl am Arbeitsplatz als auch bei wissenschaftlichen Konferenzen können wesentlich dazu beitragen, die Vereinbarkeit von Beruf und Familie zu verbessern. Es bedarf Rahmenbedingungen, die es Männern und Frauen gleichermaßen ermöglichen, berufliche und familiäre Verpflichtungen besser zu vereinbaren. Darüber hinaus könnten technische Anpassungen, wie die Entwicklung ergonomisch auf die physischen Bedürfnisse von Frauen abgestimmter Endoskope sowie der Einsatz von Strahlenschutzvorrichtungen in Kombination mit umfassenden Informationsangeboten zum Thema Strahlenschutz sowie den endoskopischen Möglichkeiten während einer Schwangerschaft 39 , zusätzliche Barrieren abbauen. Diese Maßnahmen allein sind jedoch nicht ausreichend. Erfolgreiche Modelle aus Skandinavien basieren auf Hybridansätzen, die eine verbindliche Frauenquote als Basis mit freiwilligen Programmen kombinieren. Diese Konzepte führten zu einem signifikanten Anstieg des Frauenanteils in Führungspositionen sowie in der Weiterbildung zur *advanced endoscopy* . Zudem werden verpflichtende Teilzeitoptionen angeboten. Verpflichtende Teilzeitoptionen bezeichnen institutionell verankerte Modelle, die einen gleichwertigen Zugang zu Weiterbildung, Führung und Karriereentwicklung unabhängig vom Beschäftigungsumfang sicherstellen und systematisch mit Leadership-Trainings verknüpft sind, um die kontinuierliche Karriereförderung zu gewährleisten 14 . Dual Career Paths ermöglichen beiden Geschlechtern eine parallele Facharztausbildung bei gleichzeitiger Elternzeit 38 . Weiterhin können transparente und faire Auswahlverfahren, beispielsweise durch anonyme Bewerbungsverfahren, nachweislich die Zahl weiblicher Teilnehmerinnen in *advanced endoscopy* -Programmen erhöhen 15 .

Ein weiterer zentraler Aspekt ist der Mangel an spezifischen weiblichen Mentoren und Identifikationsfiguren, der Frauen von der Wahl spezieller Fachrichtungen und Karrierewege abhalten kann. Mentorenprogramme und Frauennetzwerke sind daher essenziell, um Frauen zu ermutigen, zu unterstützen und Diversität zu fördern. So konnte beispielsweise durch Programme der *Association of Women Surgeons* und des *American College of Surgeons* der Anteil weiblicher Teilnehmerinnen in allgemein-chirurgischen Ausbildungsprogrammen von 14% im Jahr 2001 auf 40% im Jahr 2017 gesteigert werden 26 . Zur weiteren Förderung der Geschlechtergerechtigkeit sollten gezielt Förderprogramme wie Forschungsstipendien für Gastroenterologinnen oder Mentoring-Initiativen mit erfahrenen Endoskopikerinnen etabliert werden 6 15 .

Ein fundamentaler Schritt zur Erreichung einer geschlechterübergreifenden Gleichberechtigung muss jedoch bereits im Kindesalter ansetzen. Dazu gehört die bewusste Auseinandersetzung mit und das Aufbrechen tradierter geschlechtsspezifischer Rollenbilder und Stereotype, die in unserer Gesellschaft früh verankert werden, etwa durch die Auswahl von Kinderspielzeug oder Medienrepräsentationen. Es gilt, Aufklärung und Sensibilisierung zu fördern und ein Bewusstsein zu schaffen, dass solche Stereotype erheblichen Einfluss auf Berufswahl und die Berufsausübung haben. Unterstützt werden sollten Projekte, die Kinder und Jugendliche dazu ermutigen, Interessen und Berufe jenseits traditioneller Geschlechterrollen zu entwickeln. Nur durch eine Kombination dieser Maßnahmen – von der frühen Sozialisation über strukturelle Anpassungen bis hin zu gezielter Förderung und Mentoring – kann eine wirkliche Gleichstellung auf allen Ebenen der medizinischen Karriere erreicht werden.

Als Limitation der Studie ist zu beachten, dass lediglich 9,30 % der DGVS Mitglieder an der Umfrage teilnahmen. Dies birgt einen potenziellen Selektionsbias, da insbesondere Personen mit ausgeprägten Einstellungen zur Geschlechtergerechtigkeit eine höhere Teilnahmewahrscheinlichkeit aufweisen könnten. Die Geschlechterverteilung der Befragten entspricht jedoch der der Gesamtmitglieder der DGVS, sodass von einer repräsentativen Stichprobe ausgegangen werden kann. Eine weitere Limitation besteht darin, dass keine Validierung des verwendeten Fragebogens erfolgte.

Diese Studie stellt die erste deutschlandweite Untersuchung dar, die unter Gastroenterologen und Gastroenterologinnen eine detaillierte Analyse geschlechtsspezifischer Unterschiede in der endoskopischen Tätigkeit sowie der subjektiv wahrgenommenen Barrieren und Herausforderungen bietet. Die Identifikation dieser Hindernisse bildet die Grundlage für die Entwicklung zielgerichteter Interventionen, die sowohl auf individueller als auch auf institutioneller und gesellschaftlicher Ebene ansetzen können.

## References

[1] Medizinische Versorgung Die Medizin ist weiblich Dtsch Arztebl International 2024 121 8577

[2] (BLK) B-L-KfBuF Frauen in der Medizin – Ausbildung und berufliche Situation von Medizinerinnen 2004

[3] Laumann V Warum die Medizin weiblicher werden muss 2024

[4] DAdNLeVNAdW Frauen in der Wissenschaft: Entwicklungen und Empfehlungen 2022

[5] Ärztinnen holen auf. Dtsch Arztebl International 2025; Heft 5

[6] Czypionka TSF Koisser L Riedel M Gastroenterologische und hepatologische Versorgung in Österreich 2022

[7] Bryant LD Burkinshaw P House AO Good practice or positive action? Using Q methodology to identify competing views on improving gender equality in academic medicine BMJ open 2017 7 e015973 10.1136/bmjopen-2017-015973 PMC5629690 28830870

[8] Manthopoulou E Catalan-Serra I Corsetti M Gastroenterology Training and Career in Women: Challenges and Opportunities United European gastroenterology journal 2025 13 650 655 10.1002/ueg2.12764 39949291 PMC12090833

[9] Venezia L Labarile N Maselli R Women in Gastroenterology: What Is the Current Situation? Results of an Italian National Survey Digestive diseases and sciences 2024 69 1990 1995 10.1007/s10620-024-08407-8 38637458 PMC11162355

[10] Pellisé M Ebigbo A van Herwaarden YJ Diversity, equity, and inclusion in gastrointestinal endoscopy: European Society of Gastrointestinal Endoscopy Position Statement Endoscopy 2024 56 870 881 10.1055/a-2399-3226 39322023

[11] Ciacci C LG Testoni PA Gender equality in medicine: What do gastroenterologists from Italy think of it? Dig Liver Dis Off J Ital Soc Gastroenterol Ital Assoc Study Liver 2018 50 725 727 10.1016/j.dld.2018.04.006 29866629

[12] Jawaid N Boctor M LoMonaco J Canadian Gastroenterology Career Pathway Experiences: Exploring the Gender Divide Journal of the Canadian Association of Gastroenterology 2022 5 177 183 10.1093/jcag/gwac002 35919764 PMC9340613

[13] Jamorabo DS Chen R Gurm H Women remain underrepresented in leadership positions in academic gastroenterology throughout the United States Ann Gastroenterol 2021 34 316 322 10.20524/aog.2021.0597 33948055 PMC8079863

[14] Bertram KSK Ludwig S Strunz D Positionspapier – Frauen in den Gesundheitsberufen. Women in Global Health 2020

[15] Yu JX Berzin TM Enestvedt B Gender disparities in advanced endoscopy fellowship Endoscopy international open 2021 9 E338 e342 10.1055/a-1311-0899 33655031 PMC7892265

[16] Maike Busson-Spielberger MG Miemietz B Ergebnisse der Umfrage zur Erfassung der Parität von Ärztinnen in Führungspositionen und Gremien in Deutschland, Österreich und der Schweiz. DGHO Deutsche Gesellschaft für Hämatologie und Medizinische Onkologie e V 2022

[17] Sevilay Huesman-Koecke CF Frauen in der Gesundheitswirtschaft 2020

[18] Jones E Sharma S Heisler C Perceived Barriers to Professional Equality Among Women in Gastroenterology Journal of the Canadian Association of Gastroenterology 2022 5 226 233 10.1093/jcag/gwac023 36196275 PMC9527661

[19] Banchefsky S Park B Negative Gender Ideologies and Gender-Science Stereotypes Are More Pervasive in Male-Dominated Academic Disciplines Social Sciences 2018 7 27

[20] Alers M van Leerdam L Dielissen P Gendered specialities during medical education: a literature review Perspectives on medical education 2014 3 163 178 10.1007/s40037-014-0132-1 24980516 PMC4078047

[21] Stock J Frauen werden vorgeführt, Fehler werden ihnen stärker nachgetragen. Deutscher Ärztinnenbund e.V 2024

[22] Shukla R Current Challenges Facing Women in Gastroenterology: How Do We Move Forward? ACG Case Rep J 2016 3 144 145 10.14309/crj.2016.28 27144183 PMC4843135

[23] Perez YV Kesselman A Abbey-Mensah G A Glance at Gender-Specific Preferences Influencing Interventional Radiology Selection J Vasc Interv Radiol 2016 27 142 1.43E143 10.1016/j.jvir.2015.09.009 26723924

[24] Harvin G Review of musculoskeletal injuries and prevention in the endoscopy practitioner Journal of clinical gastroenterology 2014 48 590 594 10.1097/mcg.0000000000000134 24798940 PMC4222979

[25] Rembacken BJ Dixon S Albuquerque A Barriers and bias standing in the way of female trainees wanting to learn advanced endoscopy United European gastroenterology journal 2019 7 1141 1145 10.1177/2050640619877603 31662873 PMC6794690

[26] Abelson JS Chartrand G Moo TA The climb to break the glass ceiling in surgery: trends in women progressing from medical school to surgical training and academic leadership from 1994 to 2015 American journal of surgery 2016 212 566 INF 10.1016/j.amjsurg.2016.06.012 27649976

[27] Granato CM Kaul V Kothari T Career prospects and professional landscape after advanced endoscopy fellowship training: a survey assessing graduates from 2009 to 2013 Gastrointestinal endoscopy 2016 84 266 271 10.1016/j.gie.2015.08.078 26375436

[28] Carr PL Raj A Kaplan SE Gender Differences in Academic Medicine: Retention, Rank, and Leadership Comparisons From the National Faculty Survey Academic medicine : journal of the Association of American Medical Colleges 2018 93 1694 1699 10.1097/acm.0000000000002146 29384751 PMC6066448

[29] Jolly S Griffith KA DeCastro R Gender differences in time spent on parenting and domestic responsibilities by high-achieving young physician-researchers Ann Intern Med 2014 160 344 353 10.7326/m13-0974 24737273 PMC4131769

[30] Salonen E Kindergartenersʼ gender roles 2010 10.25365/THESIS.11446

[31] Obioma IF Şahin MN Rumeli Y Gender, Self-Stereotyping, and Life Goals Predict Career Interest in Germany, Nigeria, and Turkey Sex Roles 2025 91 39 10.1007/s11199-025-01579-1

[32] Tremmel M Wahl I Gender stereotypes in leadership: Analyzing the content and evaluation of stereotypes about typical, male, and female leaders Frontiers in Psychology 2023; Volume 14 –2023 10.3389/fpsyg.2023.1034258 PMC9912935 36777214

[33] Kolehmainen C Brennan M Filut A Afraid of being “witchy with a ‘b’”: a qualitative study of how gender influences residents’ experiences leading cardiopulmonary resuscitation Academic medicine : journal of the Association of American Medical Colleges 2014 89 1276 1281 10.1097/acm.0000000000000372 24979289 PMC4146658

[34] Fassiotto M Hamel EO Ku M Women in Academic Medicine: Measuring Stereotype Threat Among Junior Faculty Journal of women’s health (2002) 2016 25 292 298 10.1089/jwh.2015.5380 PMC4790213 26555562

[35] Marchant A Bhattacharya A Carnes M Can the Language of Tenure Criteria Influence Women’s Academic Advancement? Journal of women’s health (2002) 2007 16 998 1003 10.1089/jwh.2007.0348 17903076

[36] Varadarajulu S Petruff C Ramsey WH Patient preferences for gender of endoscopists Gastrointestinal endoscopy 2002 56 170 173 10.1016/s0016-5107(02)70173-9 12145592

[37] Wallis CJD Jerath A Aminoltejari K Surgeon Sex and Long-Term Postoperative Outcomes Among Patients Undergoing Common Surgeries JAMA surgery 2023 158 1185 1194 10.1001/jamasurg.2023.3744 37647075 PMC10469289

[38] Die besten europäischen Länder, um als Frau im Gesundheitswesen zu arbeiten https://www.lenstore.de/forschung/frauen-im-gesundheitswesen/

[39] Lenzen H Welsch L Reichermeier S Endoscopy during pregnancy Zeitschrift fur Gastroenterologie 2025 63 1143 1148 10.1055/a-2677-1877 41213539 PMC12602060

